# Promising bioactive compounds from the marine environment and their potential effects on various diseases

**DOI:** 10.1186/s43141-021-00290-4

**Published:** 2022-01-26

**Authors:** Akash Karthikeyan, Abey Joseph, Baiju G. Nair

**Affiliations:** 1 School of Biotechnology, National Institute of Technology Calicut, Calicut, Kerala India; 2 Nanomedical Engineering Laboratory, Riken, Wako, Saitama Japan

**Keywords:** Secondary metabolites, Marine natural products, Bioactive compounds, Novel drugs

## Abstract

**Background:**

The marine environment hosts a wide variety of species that have evolved to live in harsh and challenging conditions. Marine organisms are the focus of interest due to their capacity to produce biotechnologically useful compounds. They are promising biocatalysts for new and sustainable industrial processes because of their resistance to temperature, pH, salt, and contaminants, representing an opportunity for several biotechnological applications. Encouraged by the extensive and richness of the marine environment, marine organisms’ role in developing new therapeutic benefits is heading as an arable field.

**Main body of the abstract:**

There is currently much interest in biologically active compounds derived from natural resources, especially compounds that can efficiently act on molecular targets, which are involved in various diseases. Studies are focused on bacteria and fungi, isolated from sediments, seawater, fish, algae, and most marine invertebrates such as sponges, mollusks, tunicates, coelenterates, and crustaceans. In addition to marine macro-organisms, such as sponges, algae, or corals, marine bacteria and fungi have been shown to produce novel secondary metabolites (SMs) with specific and intricate chemical structures that may hold the key to the production of novel drugs or leads. The marine environment is known as a rich source of chemical structures with numerous beneficial health effects. Presently, several lines of studies have provided insight into biological activities and neuroprotective effects of marine algae, including antioxidant, anti-neuroinflammatory, cholinesterase inhibitory activity, and neuronal death inhibition.

**Conclusion:**

The application of marine-derived bioactive compounds has gained importance because of their therapeutic uses in several diseases. Marine natural products (MNPs) display various pharmaceutically significant bioactivities, including antibiotic, antiviral, neurodegenerative, anticancer, or anti-inflammatory properties. The present review focuses on the importance of critical marine bioactive compounds and their role in different diseases and highlights their possible contribution to humanity.

## Background

Natural products have been used for the treatment of human ailments since the beginning of mankind. Ocean remains as one such treasure for natural products. The oceans cover more than three-quarters of the earth’s surface and harbor most of the planet’s diversity. But the marine biotope, which is still an unexplored area, can provide us with rich novel natural products. For decades, microbial natural products have been the reservoir for drug discovery, yet the microorganisms inhabiting the world’s oceans have largely been overlooked in this regard [1]. Microbial communities in extreme environments have immense potential as unexploited resources discovering bioactive molecules or novel drugs. Among the potential sources of natural products, bacteria have been proven to be a prolific source with a surprisingly small group of taxa accounting for the vast majority of compounds discovered [2].

Although more than 100 drugs exist today that come from terrestrial microorganisms, arguably the most important drug in medicine is the potential from land-based microbial sources, which began to dwindle nearly 10 years ago. Pharmaceutical investigators searched around the globe for new terrestrial, drug-producing microbes, but with diminishing payback [3].

The first serious effort in studying marine natural products started in 1951 with Bergman and Feeney’s pioneering work that resulted in the isolation of spongothymidine and spongouridine from the sponge *Cryptotethya crypta* Laubenfels. This finding led to the synthesis of arabinosyl cytosine (Ara-C), a marine-derived anticancer agent used mainly to treat different forms of leukemia. Since the 1950s, marine organisms have been shown to be rich sources of structurally novel and biologically active metabolites, constituting valuable opportunities for drug discovery, an area of extreme importance among the scientific community [4].

Although more than 30,000 diseases have been clinically described, less than one-third of these can be treated symptomatically, and only a few can be cured. New therapeutic agents are needed to treat medical needs that are currently unmet. Natural products once played a major role in drug discovery. The marine environment coves more than 70% of the world’s surface. In the past, this has proven to be a rich source of extremely potent compounds, which represent a considerable number of drug candidates [4]. However, to date, the biodiversity of marine microbes and the versatility of their bioactive metabolites have not been fully explored.

The marine environment was once thought to have high salt, poor nutrition, and less microbial growth. On the contrary, soil microbes are widely regarded as living in a more crowded and competitive environment. The ecology of marine natural products reveals that many of the compounds isolated from the marine source are chemical weapons and have evolved into highly potent inhibitors of biological processes in the prey, predators, or competitors of the marine organisms that utilize them for survival [5].

## Main text

### Introduction

Marine sources have played a significant role as an origin for lead molecules ascertained for various pharmacological utilizations in recent times. Interestingly, marine microorganisms remain as the most undiscovered and essential provenience of umpteen bioactive metabolites. From the shallow water in the seashore to the abysmal seaward areas that canvas 70% of the biosphere, microorganisms engross an endurable stretch [6]. The varying temperature, pressure, and source of light in the marine system compared to the terrestrial environment possibly helps in producing novel secondary metabolites by some marine organisms.

**Fig. 1:**
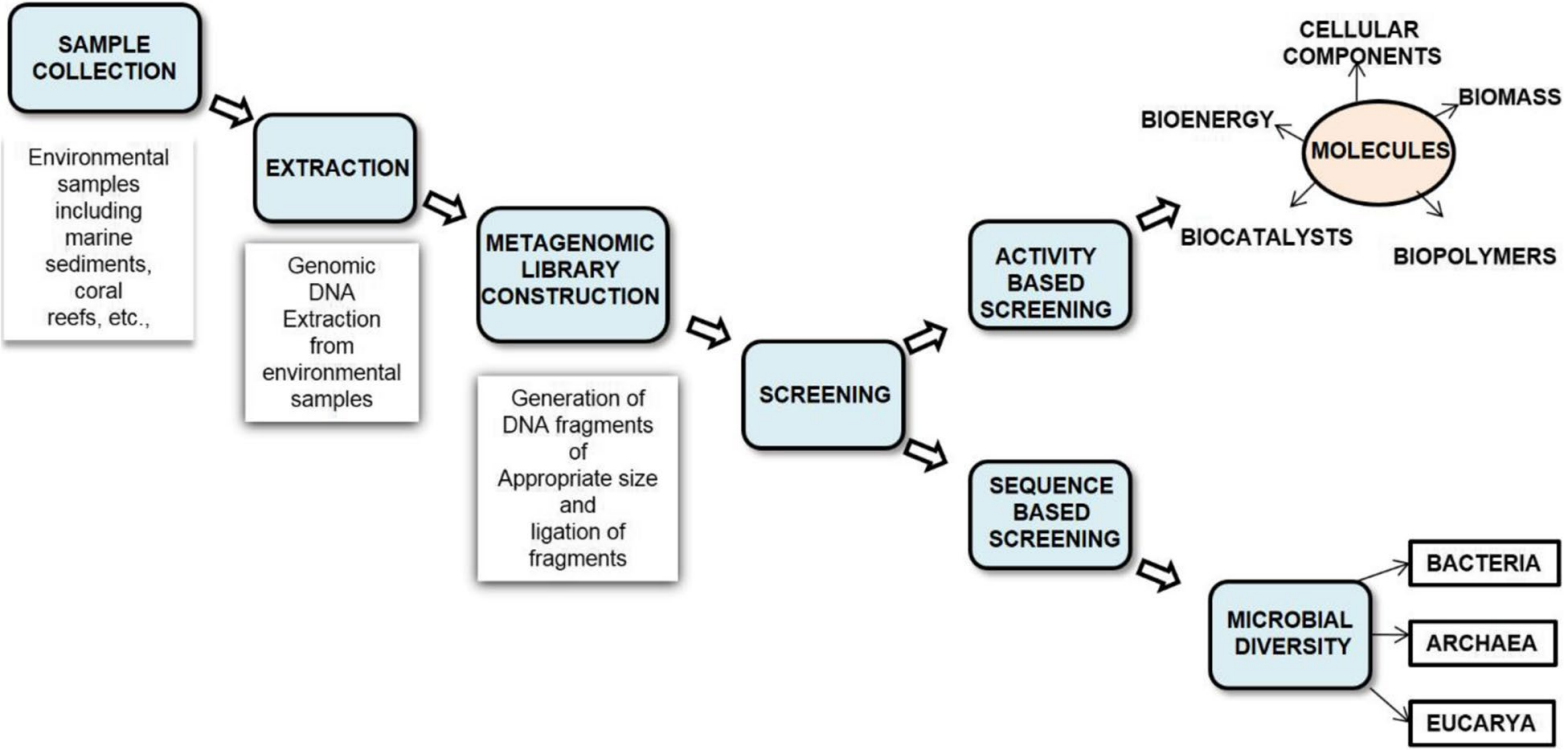
Sample collection and processing by a metagenomic approach (Marine environmental samples are collected from different marine sources, and the genomic DNA is extracted from the samples. The metagenomics library construction helps in the generation of DNA fragments of appropriate size and also in the ligation of the fragments followed by screening)

Microbes, especially in the marine environment, can withstand high salt concentrations, high pressure, nutrition depletion, and cold temperatures. Natural sources producing biological materials, screened by high throughput screening methods for their therapeutic activity, lead to developing a commercially viable process or product [7]. Bioprospecting marine habitat is one of the most prolific platforms because of its diverse and under passed microbial population. Microbes can easily detect, adapt, and react to their environment and compete by producing specific secondary metabolites for protection and survival. These compounds developed in reaction to stress have shown value in biotechnological or pharmaceutical applications [7] (Fig. 1).

In reality, marine natural products’ ecology shows that many of these compounds are chemical weapons and have grown into highly potent physiological process inhibitors in prey, predators, or marine organism rivals that use them for survival. Bioprospecting will help in unraveling the enigma of the bioactive metabolites from marine microbes [8].

From the beginning of humankind, natural products have been a beneficial source as a remedy for various ailments. In worldwide, the available drugs for clinical purpose represents more than 50% are of their natural origin. The drug discovery process from natural products is still ongoing due to synthetic drugs’ side effects [9]. The crude product has a significant impact on producing new medicines that bypass infectious diseases [10].

**Fig. 2:**
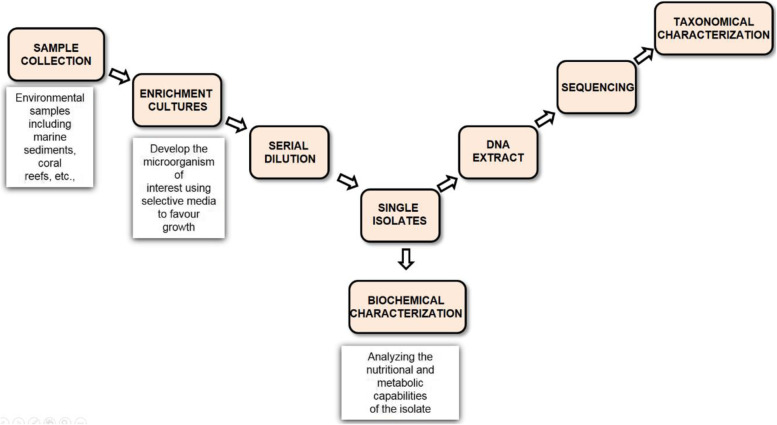
Sample collection and processing by culture-dependent approach (In the culture-dependent method, the microorganisms are enriched using selective media followed by biochemical characterization and taxonomical characterization.)

The marine microbial species tends for conceivable biotechnological and is also an essential source of ecological maintenance. It is evident from the 16S rRNA sequencing that marine microbial species such as Bacteria and Archae have a highly diverse taxonomy [11]. Metagenomic studies have revealed that extremophile prokaryotes from marine habitats are also sources of novel genes and, consequently, new bioproducts, including enzymes and other active metabolites [12] (Fig. 2).

The extreme ecological variations in the marine habitat forced the inhabitant organisms to produce a class of tolerable hydrolase enzymes such as proteases, lipases, glycoside hydrolases, which is used in industrial processes due to their novel specificities and properties of tolerance to extreme industrial conditions. Therefore, studying and understanding these microorganisms is necessary to exploit the biochemical, ecological, evolutionary, and industrial potential [13].

### Bioactivity of novel compounds from marine microorganisms

The resistance of microorganisms against antibiotics is a severe global issue. There is a need for novel chemical compounds capable of a battle against infections provoked by multidrug-resistant pathogens. The discovery of new products from natural sources is mainly essential for the development of novel antimicrobial agents. Currently, antimicrobial drugs for medical treatment derived from natural origin exhibit Actinobacteria as the most important secondary metabolite source. Carbohydrates, pigments, polyphenols, peptides, proteins, and essential fatty acids are marine bioactive compounds widely studied for various applications. These compounds have rheological effects and are found helpful in the food industry and diverse biological functions such as antioxidant, anti-thrombotic, anti-coagulant, anti-inflammatory, anti-proliferative, anti-hypertensive, anti-diabetic, and cardio-protective activities [14]. Novel bioactive compounds with extensive activities will be discussed here.

#### Antibacterial potential of bioactive compounds from marine microorganisms

The treatment options for some diseases like Alzheimer’s disease, Parkinson’s disease, rheumatoid arthritis (RA) and other forms of arthritis, type-1 diabetes, heart diseases, irritable bowel syndrome, allergies, asthma, cancer, and many others are limited, and certain drugs have significant side effects on patients’ health on overdose. Therefore, other alternatives that could theoretically help to manage these troublesome bacterial infections need exhaustive investigations. Since ancient times, the utility of natural products for antimicrobial therapy and other diseases has been a promising treatment [14]. The antibacterial potential of specific bioactive compounds from marine bacteria is extensively mentioned below.

##### Spirotetronate compounds

Maklamicin of the class polyketide is a novel spirotetronate compound isolated from the *Micromonospora* sp. GMKU326 in Thailand. Maklamicin exhibited potent antimicrobial activity with MIC values of 0.2, 1.7, 6.5, 13, and 13 μg/ml against *Micrococcus luteus*, *Bacillus subtilis*, *Bacillus cereus*, *Staphylococcus aureus*, and *Enterococcus faecalis*; on the other hand, it showed a lower activity against *Candida albicans* (MIC = 50 μg/ml). Maklamicin also showed a potent cancer cell cytotoxicity [15].

The *Actinomadura* sp. TP-A0878 is capable of producing a spirotetrone compound nomimicin of polyketide origin. Nomimicin showed potent antimicrobial activity against *Micrococcus luteus*, *Candida albicans*, and *Kluyveromyces fragilis* with MIC values of 6.3, 12.5, and 12.5 μg/ml [16].

Lobophorin F isolated from the *Streptomyces* sp. SCSIO 01127 is a novel compound possessing antibacterial and antitumor activities with MIC values of 2,8,8 μg/ml against *Bacillus thuringiensis*, *Staphylococcus aureus*, and *Enterococcus faecalis* [17]. The *Streptomyces* sp. strain MS1 00061 with provenance from the South China Sea is efficient to produce three secondary metabolites of the family lobophorin (lobophorin A, B, and G). The significant anti-BCG effect is identified with these three metabolites [18].

##### Ansamycin-type polyketide compounds

Novel ansamycin-type compounds isolated from Chilean Atacama Desert soil from the *Streptomyces* sp. strain C34 labeled as chaxamycins A–D showed potent antibacterial activity against *Staphylococcus aureus* ATCC25923 and *Escherichia coli* ATCC25922. Chaxamycins (A–C) were found to inhibit ATPase activity (41–46% of inhibition at 100 micromolar) [19].

##### Βeta-diketones, aromatic compounds

*Streptomyces asenjonii* KNN 42.f from Northern Chile produced novel bioactive compounds of the beta-diketones family. Asenjonamide C showed the highest antibacterial activity with MIC 1.8 μg/ml, 3.9 μg/ml, and 5.4 μg/ml against methicilin-sensitive *Staphylococcus aureus, Enterococcus faecium,* and *Escherichia coli* [20].

Gilvocarcin HE isolated from the *Streptomyces* sp. QD01-2 is termed to exhibit antimicrobial activity against *Staphylococcus aureus, Bacillus subtilis, Escherichia Coli,* and *Candida albicans*. Cytotoxic activity against the MCF-7, K562, and P388 cell lines, with IC_50_ values of 36, 39, and 45 μM convinced that the vinyl side chain increased the cytotoxicity and antimicrobial activities [21].

Zunyimycins B and C isolated from the *Streptomyces* sp. FJS31-2 exhibited antimicrobial activity with MIC = 0.94 μg/ml and MICs between 3.75–8.14 μg/ml against MRSA isolates [22].

##### Tetracenediones

*Streptomyces formicae* KY5 strains can produce polyketides formicamycins A–L, efficient to inhibit MRSA with MIC 0.41 μg/ml and vancomycin-resistant *Enterococcus faecium* (VRE) with MIC 0.82 μg/ml [23].

##### Lactones

Allocyclinones produced from the *Actinoallomurus* sp. ID145698 exhibited antibacterial activity with MIC range of 0.25–0.5 μg/ml against *Staphylococcus aureus, Streptococcus pyogenes,* and *Enterococcus faecalis* whereas *Enterococcus faecium* showed MIC = 4 μg/ml. The number of substituents regulated the increase in antibacterial activity [24].

RSP 01 from the actinomycin group is a bicyclic chromopeptide lactone biosynthesized with RSP02 by the *Streptomyces* sp. RAB12. RSP01 with higher antimicrobial potential is possessed to have a ketocarbonyl group with MIC values of the range 0.007 to 0.06 μg/ml [25].

##### Quinolones

*Agelas oroides*, a marine sponge produced a novel chlorinated quinolone, ageloline A, which can inhibit the growth of *Chlamydia trachomatis* inclusion with an IC_50_ value of 2.1 μg/ml. Ageloline A lowered the genomic damage activated by an oxidative stress inducer, 4-nitroquinoline-1-oxide [26].

##### Xanthones

An alluring bioactive compound buanmycin isolated from a tidal mudflat in Buan (Republic of Korea) efficient with MICs 0.42–12.5 μg/ml against Gram-positive (*Staphylococcus aureus, Bacillus subtilis, Kocuria rhizophila*) and Gram-negative bacteria (*Salmonella enterica, Proteus hauseri*) and able to obstruct *Staphylococcus aureus* sortase A with an IC_50_ value of 43.2 μM [27].

Liu et al. isolated four bioactive compounds citreamicin A, citreamicin B, citreaglycon A, and dehydrocitreaglycon possessing antibacterial activity against *Staphylococcus haemolyticus, Staphylococcus aureus, and Bacillus subtilis*. Because of the five-member nitrogen heterocycle presence in their structure, citreamicin A and citreamicin B were more active [28].

##### Peptides

Kocuriapalustris F-276,345 produced a novel thiazozyl peptide kocurin (PM181104) for medication of Gram-positive bacterial infections by blocking its protein biosynthesis at the translation stage. Further studies have shown that organ and systemic infections in mice can be minimized due to kocurinin [29].

##### Terpenoids

Three novel meroterpenoids—napyradiomycins, analogs isolated from the *Streptomyces* sp. strain SCSIO 10428 (Beihai, Guangxi province, China). 3-dechloro3-bromonapyradiomycin A1 are effective against *Staphylococcus aureus*, *Bacillus subtilis,* and *Bacillus thuringensis* and revealed cytotoxic activity against human cancer cell lines [30].

A novel actinomadurol isolated from Actinomadura KC191 afforded a novel scaffold for antibiotic diagnosis due to its unique 19-norditerpenoid-carbon. It inhibited *Bacillus subtilis, Staphylococcus aureus, Kocuria rhizophila, Proteus hauseri, Salmonella enteric* with MIC values of 0.39 to 3.12 μg/ml [31].

##### Lipopeptides

Arylomycin A6 identified from parvus HCCB10043 exhibited antibacterial activity with the MIC of 1 μg/ml against *Staphylococcus epidermidis* HCCB20256 with the requirement of ultra-performance liquid chromatography coupled with tandem quadrupole and time of flight high-resolution mass spectrometry [32].

##### Depsipeptides

A *Streptomyces* sp. capable of producing ohmyungsamycins A and B containing unusual amino acid units showed inhibitory activity against *Bacillus subtilis, Kocuria rhizophila,* and *Proteus hauseri* with MICs = 1.56–49.5 μg/ml [33].

Sun et al. identified compounds active against different MRSA strains fijimycins A and C, with MICs in the range of 4–32 μg/ml from the *Streptomyces* sp. CNS-575 strain which belongs to the etamycin-class depsipeptides [34].

##### Amylolytic actinobacterium

The mangrove ecosystem, due to its varied microbial association, tends to produce unique bioactive compounds. *Microbacterium mangrovi* MUSC 115T, Sinomonashumi MUSC 117T, and *Monashia flava* MUSC 78T belonging to actinobacteria, were isolated from mangrove soils at Tanjung Lumpur, Peninsular Malaysia. The extracts *Microbacterium mangrovi* MUSC 115T, Sinomonashumi MUSC 117T, and *Monashia flava* MUSC 78T exhibited bacteriostatic effects bacteria such as Methicillin-resistant Staphylococcus aureus (MRSA) ATCC 43300, ATCC 70069, Pseudomonas aeruginosa NRBC 112582. The neuroprotective studies revealed *M. mangrovi* MUSC 115T extract can exhibit neuroprotective properties in oxidative stress and dementia model. The extract *M. flava* MUSC 78T defended SHSY5Y neuronal cells in the hypoxia model. Anti-cancer effects by the extracts *M. mangrovi* MUSC 115T and *M. flava* MUSC 78T against Ca Ski cell line make the compound more alluring [35].

#### Antioxidant potential of bioactive compounds from marine microorganisms

Marine sediments acquired from Chennai, Tamilnadu, India, labeled as VSKB 1 to VSKB 6 were screened out for their antibacterial and antioxidant activities in which VSKB 3 exhibited activity against *Salmonella typhi* and higher antioxidant activity in DPPH scavenging assay (88.32%), metal chelating assay (80.7%), and reducing power assay (0.80%) VSKB-3. Further, the isolate VSKB-3 is partially characterized by conventional methods, using the Nonomura key. It showed similar characteristics to *Streptomyces bluensi* and will be helpful in producing drugs against *Salmonella typhi* [36].

#### Anti-larvicidal potential of bioactive compounds from marine microorganisms

(Z)-1-((1-hydroxypenta-2,4-dien-1-yl)oxy) anthracene-9,10-dione extracted from *Nocardia alba* KC710971 was analyzed for its anti larvicidal activity in different concentrations against mosquito larvae *Aedes aegypti, Culex quinquefasciatus,* and *Anopheles stephensi* and also Newcastle disease virus and infectious Bursal disease virus. Similar reports were acquired by Vijayakumar et al. [37] and Subhasish Saha et al. [38, 39] in the *Nocardiopsis* sp. Dhanasekaran et al. identified actinomycetes strains having larvicidal activity against *Anopheles* mosquitoes [40]. The novel bioactive substances present in the bacteria help destroy the larvae’s cuticle layer, thereby inhibiting it [41].

#### Anti-inflammatory activity of bioactive compounds from marine microorganisms

Inflammation, a crucial component of host responses to multiple stimuli, including injury, microbial invasion, and immune responses, includes different biological pathways guided by external and internal stimuli. Compounds known as non-inflammatory agents may be modulated, diminished, or blocked by these biological pathways. Drugs developed from natural products are in high demand as the synthetic drugs used in treating inflammatory disorders cause adverse side effects. Novel compounds like sesquiterpenoids, diterpenes, steroids, polysaccharides, alkaloids, and fatty acids, isolated from marine organisms, are found to exhibit anti-inflammatory activity.

##### Polysaccharides

Marine polysaccharides including alginate, porphyran, fucoidan, chitin, and chitin derivatives, are used as down regulators of allergic responses [42]. Polysaccharides isolated from algae that are mostly sulfated exhibit anti-inflammatory activity in vitro and in vivo [43–45], which attributes to their structure and physicochemical characteristics [46].

##### Proteins

Marine lectins are found to have anti-inflammatory activity due to their carbohydrate-binding site [47]. Green seaweed *Caulerpa cupressoides* efficiently produce lectin and is administered in the left temporomandibular joint half an hour before zymosan injection. As a result, reduced zymosan-elicited arthritis and mechanical hypernociception are noticed in rats. Also, suppression in the leukocyte accumulation in synovial fluid is observed. But when treated with opioid receptor antagonist naloxone or ZnPP-IX, the activity of lectin declined. However, lectin blocked leukocyte influx and TNF-alpha and IL-1beta expression in the temporomandibular joint, proving that lectin vitiates temporomandibular joint hypernociception and inflammation depends partially on suppression of IL-1beta and TNF-alpha [48].

#### Enzyme inhibitors

Polymeric 3-alkylpyridinium salts composing of N-butyl (3-butylpyridinium) have been isolated from marine sponge Renierasarai. N-Butyl-3-butylpyridinium iodide, the monomer of the inhibitor, has been synthesized which acts as acetylcholinesterase inhibitors. The TLC bioautography method was carried out to assess the acetylcholinesterase inhibitory activity of the marine extracts. Extracts obtained from soft corals were more active. 14-Acetoxycrassine was determined as the bioactive compound using X-ray diffraction. Adding to this, the acetylcholinesterase inhibitory activity of 14 cembranoids has been isolated from soft corals *Euniceaknighti* and *Pseudoplexauraflagellosa*. The quantitative test, 14-acetoxycrassine and asperdiol, exhibited IC_50_ values of 1.40–0.113 and 0.358–0.130 μM, respectively [49].

In Alzheimer’s disease, acetylcholinesterase inhibition is an important checkpoint. Acetylcholinesterase, alphaglucosidase, and xanthine oxidase inhibitory activity of 55 ethyl acetate extracts were identified in which *Vibrio neocaledonicus* exhibited 98.95% activity [50].

Table 1 shows the bioactive secondary metabolites isolated from marine sources, their structure, and applications in different fields.

**Table 1:** Bioactive secondary metabolites from marine sources

	Secondary metabolites from marine sources					
SN	Secondary metabolites	Species	Structure	Applications	Pubchem ID	Reference
	**Anticancer**					
1	Aureoverticillactam	*Streptomyces aureoverticillatus*		Cytotoxicity of various cell types of tumors	9868536	[51]
2	Caprolactones	*Streptomyces* sp.		Activity against cancer cell lines	10401	[52]
3	Chinikomycins	*Streptomyces* sp.		Antitumor action against different cancer cell lines in humans	11273076	[53]
4	IB-00208	*Actinomadura* sp.		Cytotoxic activity on tumor cell lines and bactericidal activity against Gram-positive bacteria	139583280	[54]
5	Salinosporamide A (NPI-0052)	*Salinisporatropica*		Cytotoxicity, inhibition of the proteosome and inhibition of the activation of NF-κB	11347535	[55]
6	Urdamycin	*Streptomyces fradiae*		Contains biomolecules of aminoglycoside and strong antibacterial and anti-cancer activity	443819	[56]
7	Himastatin	*Streptomyces hygroscopicus*		Includes valine, leucine, threonine, α-hydroxyisovaleric acid, 5-hydroxypiperazic acid, and a dimeric hexahydropyrroloindole.	9855348	[57]
8	Daryamide D	*Streptomyces strain CNQ-085*		Cytotoxic activity against cell line HCT-116 of human colon carcinoma and antifungal activity against Candida albicans	132609319	[58]
9	Marinomycin	*Marinispora sp. strain CNQ-140*		Inhibition of cancer cell proliferation		[59]
10	Manumycin	*Streptomyces sp. M045*		Antitumor activity against different human cancer cell lines	6438330	[53]
11	Marmycin	*Streptomyces sp.*		Cytotoxicity of tumor cells tended to correlate with moderate apoptosis induction and arrest during the G1 cell cycle process	91801297	[60]
12	Nonactin	*Streptomyces tsukubaensis, Streptomyces griseus, Streptomyces chrysomallus, and Streptomyces werraensis.*		Vigorous antineoplastic and antibacterial activity	72519	[61]
13	Chartreusin	*Streptomyces chartreusis*		Active against certain gram-positive bacteria	5281394	[62]
14	Altemicidin	*Streptomyces sioyaensis* SA-1758.		Acaricidal activity and antitumor activity	11036174	[63]
15	Streptochlorin	*Streptomyces sp.*		Promising chemotherapeutic agent to the treatment of cholangiocarcinoma	44608049	[64]
16	Marineosins	*Streptomyces sp.*		Significant inhibition of human colon carcinoma	135960042	[65]
17	Ammosamides	*Streptomyces variabilis*		Cytotoxicity to the MIA PaCa-2 pancreatic cancer cell line	25113669	[66]
18	Caboxamycin	*Streptomyces sp. NTK 937*		Inhibitory activity against Gram-positive bacteria, selected human tumor cell lines and the enzyme phosphodiesterase	135957253	[67]
19	Hoiamide D	*Symploca* sp		screening inhibitory activity in contrast to 53/Mdm2 interaction	56835050	[68]
20	Niphateolide	*Niphates olemda*		p53-Hdm2/Mdm2 interaction inhibitor	132989992	[69]
21	Hexylitaconic acid	*Arthrinium* sp		blocks p53/Mdm2 binding	11447214	[70]
22	Lissoclinidine B	*Lissoclinum cf*. *badium*		kills altered cells with wild-type p53	25147779	[71]
23	Himeic acid A	*Aspergillus* sp.		Ubiquitin-Activating Enzyme (E1) inhibitory action	11774903	[72]
24	Girolline	*Cymbastela cantharell* and *Axinella brevistyla*		initiating G2/M cell cycle arrest in cancer cells	362388	[73]
25	Leucettamol A	*Leucetta aff*. *microrhaphis*		inhibits the ubiquitin E2 enzymes Ubc13 and Uev1A by 50%	6271251	[74]
26	Dysidiolide	*Dysidea etheria*		capable of inhibiting Cdc25 protein phosphatase	11269661	[75]
27	Sulfircin	*Ircinia s*p		inhibit Cdc25 phosphatase	44381469	[76]
28	Coscinosulfate	*Coscinoderma mathewsi*		inhibitory activity towards Cdc25A	102305354	[77]
29	Halenaquinone	*Xestospongia carbonaria*		irreversible inhibitor of recombinant human Cdc25B phosphates	370346	[78]
30	Secalonic acid D	*Penicillium oxalicum*		slow the course of the cell cycle in human embryonic palatal mesenchymal cells	73431	[79]
31	Stellettin B	*Jaspis stellifera*		decrease in Cdk and an increase in p27 expression	5352082	[80]
	**Antibacterial**					
32	Abyssomicins	Verrucosispora sp.		Inhibits the pathway between chorismate and *para*-aminobenzoic acid	12094197	[81]
33	Frigocyclinone	*Streptomyces* griseus		Lead molecule against Kaposi’s Sarcoma Associated Herpesvirus KSHV	11476774	[82]
34	Gutingimycin	*Streptomyces* sp.		Antibacterial antifungal and antimicroalgal activities	136835719	[83]
35	Helquinoline	*Janibacterlimosus*		Antibacterial antifungal and antimicroalgal activities	10466080	[84]
36	Himalomycins	*Streptomyces* sp.		Antimicrobial activity against Gram-positive bacteria	11765992	[85]
37	Lajollamycin	*Streptomyces nodosus*		Antimicrobial activity against drug-sensitive and -resistant Gram-positive bacteria and inhibited the growth of B16-F10 tumor cells *in vitro*	139587457	[32]
38	Tylosin	*Streptomyces fradiae*		Potential for the treatment of respiratory and other infections caused by *Mycoplasma* species		[86]
39	Maklamicin	*Micromonospora sp.* GMKU326		Antimicrobial activity against Gram-positive bacteria	101796870	[87]
40	Lobophorin K	*Streptomyces* sp. M-207		Antibiotic activity against pathogenic Gram-positive bacteria	139590476	[88]
41	Asenjonamide C	*Streptomyces asenjonii strain KNN 42*		Antimicrobial activity against Gram-positive bacteria	139589509	[20]
42	Gilvocarcin HE	*Streptomyces* sp.		More significant cytotoxicity and antimicrobial activity due to the vinyl side chain	102439806	[21]
43	Zunyimycins	*Streptomyces* sp. FJS31-2		Inhibits the proliferation of lung cancer cells by the activation of apoptosis by an AKT pathway		[22]
44	Formicamycins A	*Streptomyces formicae*		Potent antibacterial activity against clinical MRSA and VRE isolates		[23]
45	Allocyclinones	*Actinoallomurus*		Possess activity against various Gram-positive bacteria, including antibiotic-resistant strains, with increasing antibacterial potency with the number of chlorine substitutes		[24]
46	Ageloline A	*Streptomyces sp. SBT345*		Able to reduce oxidative stress and genomic damage induced by the oxidative stress inducer 4-nitroquinoline-1-oxide (NQO)	1884	[26]
47	Buanmycin	*Streptomyces sp*		Inhibition of sortase A, which is a promising target for antibiotic Discovery		[89]
48	Citreamicin	*Streptomyces caelestis*		Citreamicins exhibit cytotoxic activity against HeLa and Hep62 cells in addition to their potent antibiotic activity	3083114	[28]
49	Kocurin	*Kocuria sp. strain MI-67-EC3-038*		Kocurin is active against methicillin-resistant *Staphylococcus aureus* (MRSA)		[29]
50	Fijimycins	*Streptomyces* sp.		Possess significant *in vitro* antibacterial activity against methicillin-resistant *Staphylococcus aureus* (MRSA) strains		[86]
51	Arylomycin	*Streptomyces sp.* HCCB10043		Inhibits a promising antimicrobial target, type I signal peptidase (SPase)		[90]
	**Antibacterial; anticancer**					
	1-Hydroxy-1-Norresistomycin	*Streptomyces variabilis*		Potent cytotoxic activity against cell lines *viz*. HMO2 (gastric adenocarcinoma) and HePG2 (hepatic carcinoma) *in vitro*		[91]
	**Antibacterial; antifungal**					
52	Bonactin	*Streptomyces* sp.		Bonactin displayed antimicrobial activity against both Gram-positive and Gram-negative bacteria as well as antifungal activity	11741721	
	**Anti-inflammatory**					
53	Diazepinomicin (ECO-4601)	*Micromonosproa* sp.		Preclinical broad-spectrum antitumor potential, antioxidant and anti-protease activities.	9868980	[92]
54	Cyclomarins	*Streptomyces* sp.		Interesting lead structures for the development of drugs against tuberculosis and malaria		[93]
	**Neurogenic activity**					
55	Komodoquinone A	*Streptomyces* sp. KS3		Induces differentiation of neuronal cells in the neuroblastoma cell line, Neuro 2A and arrests the cell cycle in step G1	11756746	[94]
56	Granaticins	*Streptomyces lateritius*		Granaticinhas significant antitumor activity against P-388 lymphocytic leukemia in mice and cytotoxicity against KB cells.		[95]
57	Hymenialdisine	*Agelasidae, Axinellidae, and Halichondriidae*		suppresses many pro inflammatory cytokines (IL-1, IL-2, IL-6, and NO) by inhibition of NF-kB signaling pathway	135413546	[96]
	**Antifungal**					
58	Amphotericin B	*Streptomyces nodosus*		Treatment of most systemic fungal infections	91819969	
	**Anti tuberculosis**					
59	Rifampicin	*-*		Rifampicin had immunomodulatory effects through its ability to modify human monocyte production of measured cytokines		[97]
60	Streptomycin	*-*		Antibiotic activity against gram-positive and gram- negative bacteria	19649	[97]
61	Amikacin	*-*		Potent activity against Antibiotic-Resistant Clinical Isolates	37768	[97]
62	Viomycin	*-*		Tuberculostatic agent active against both streptomycin – sensitive nad streptomycin – resistant strains	135565959	[97]
63	Capreomycin	*-*		Bactericidal *in vitro* among the anti-TB drugs against non-replicating tubercle bacilli	135565060	[97]
64	Kanamycin	*-*		Kanamycin intramuscular administration had a satisfactory effect in mice with staphylococci, pneumococci, and Ulebsiella pneumonia infections	6032	[97]
65	Cycloserine	*-*		Antituberculous activity *invitro* and *invivo*	6234	[97]

### Leading secondary metabolites from marine sources and their role against various diseases

#### Against tuberculosis

Tuberculosis is the greatest threat around the globe. However, there are anti-tuberculosis (anti-TB) medicines, which lowered the fatality drug-resistant forms. Nevertheless, of the clinical drugs, biodiverse marine microorganisms have been identified as a drug source in treating tuberculosis. Nearly 170 compounds isolated from marine sources tended to exhibit anti-TB properties. The current anti-TB agents rifampicin, streptomycin, amikacin, viomycin, capreomycin, kanamycin, and cycloserine possess in vitro activity against *Mycobacterium tuberculosis* with MICs of 0.2, 0.5, 1.0, 4.0, 5.0, and 6.0 μg/mL, respectively [97]. The initial MIC value should be less than 64 μg/mL to identify potential anti-TB compounds, or the growth inhibition should be more significant than 75% at 12.5 μg/mL [98, 99]. Additionally, a selectivity index (SI, IC_50_/MIC) more significant than 10 has been used as a benchmark to screen anti-TB compounds that can further develop [98, 99]. With their unique aquatic environment and rich biodiversity, the oceans have proven to be a plentiful source of diverse natural products with significant antimicrobial, antiviral, antimalarial, antitumor anti-inflammatory, and antioxidant activities [100].

#### Neurodegenerative diseases

Neurodegenerative disorders are characterized by mitochondrial dysfunction and reactive production of oxygen species (ROS), among cellular pathologies, thereby related to oxidative stress. The central nervous system is peculiarly sensitive to free radical damage due to its high oxygen consumption ratio, rich content of phospholipids, and high levels of iron, which can catalyze oxidative reactions and contribute to an increase in the production of free radicals. This is coupled with a low content of antioxidant defenses in the brain that is even more altered in Neurodegenerative disease.

##### Secondary metabolites preventing oxidative stress

Oxidative stress is a frequent checkpoint in neurodegenerative diseases, widely associated with mitochondria. These two compounds, glutathione and catalase, displayed complete protection against oxidative stress with mitochondrial function improvement, ROS production inhibition, and antioxidant enzyme levels. Further studies have reported that anhydroexfoliamycin acts as an inducer of Nrf2 nuclear translocation over the Nrf2-ARE pathway and can significantly inhibit the uncoupler’s mitochondrial effect FCCP over cytosolic Ca2+, pointing mitochondria as a cellular target for this molecule. Also, both compounds were able to reduce the caspase-3 activity induced by staurosporine, an apoptotic enhancer. These show that Streptomyces metabolites could help develop new drugs to prevent neurodegenerative disorders such as Parkinson’s and Alzheimer’s diseases and cerebral ischemia [101].

The *Streptomyces* sp. UTMC 1334 is considered a potential anti-acetylcholinesterasic sources with an IC_50_ value of 0.36 ± 0.02 μg/mL, since extracts with an IC_50_ value lower than 1.0 μg/mL were considered strong anti-acetylcholinesterasic [102, 103]. The *Streptomyces* sp. UTMC 1334 is taxonomically identified as *Streptomyces lateritius* (99.41%). This is the first report of marine-isolated *Streptomyces lateritius* producing metabolites with AChE inhibitory activity. Six antibiotics of the granaticin group have been isolated from *Streptomyces lateritius* so far. The Granaticins are a well-documented series of quinone antibiotics and are reported to have antibacterial, antitumor, and anti-protozoal activities [104, 105].

Granaticin B is highly active against *Staphylococcus aureus* with a MIC range from 0.9 to 3.6 μmol/l. Effective inhibition of biofilm formation against *Staphylococcus aureus* is also reported [95]. Streptocyclinones A and B, isolated from the *Streptomyces* sp. to improve AD hallmarks, were evaluated. Compounds were able to protect SH-SY5Y neuroblastoma cells from H_2_O_2_-induced oxidative injury by activating the nuclear factor E2-related factor (Nrf2) [106].

##### Alzheimer’s disease

**Fig. 3:**
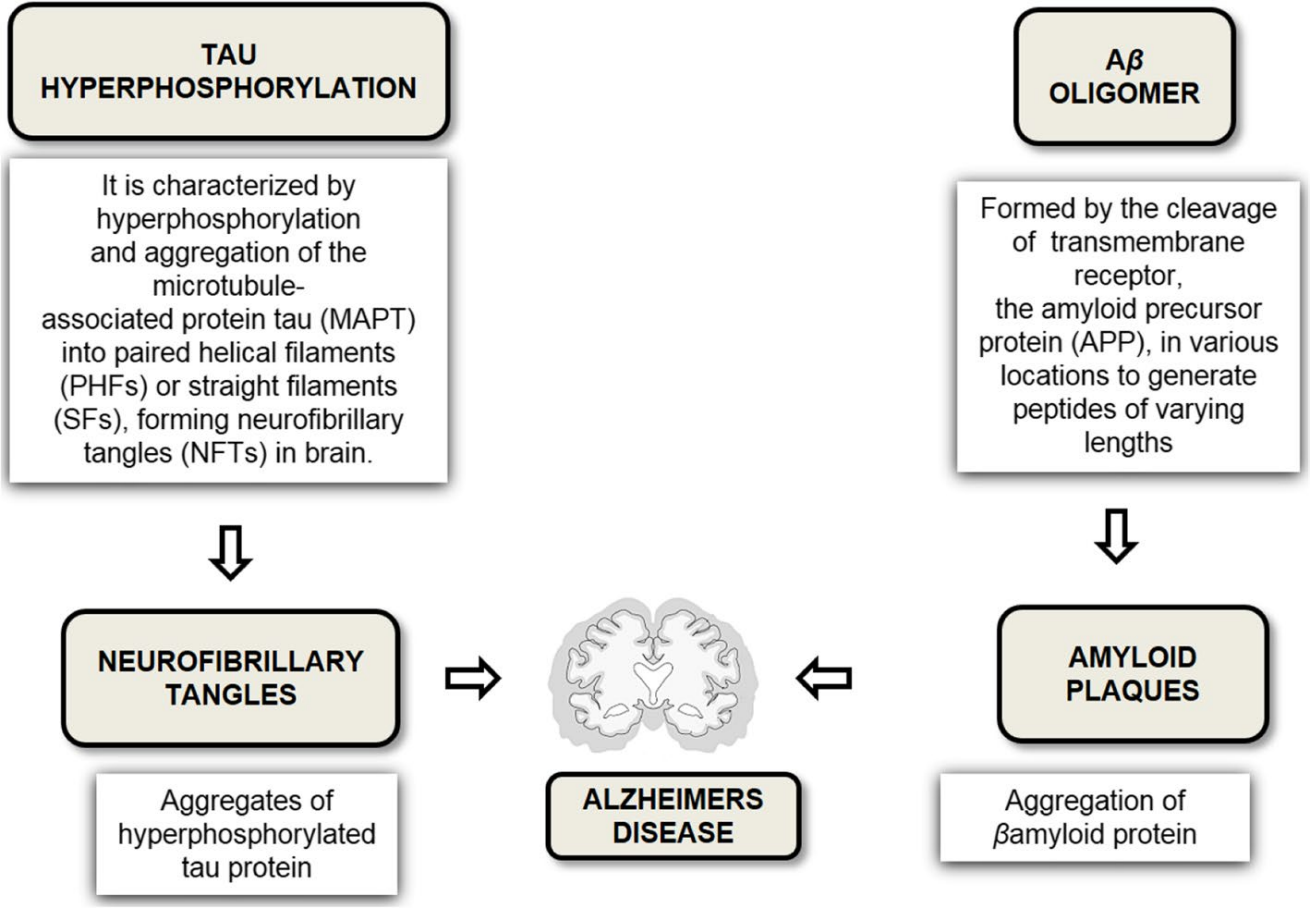
Pathological hallmarks of Alzheimer’s disease

Alzheimer’s disease (AD) is a slow and progressive degeneration with synaptic loss and final neuronal death. The impairments are located in specific brain regions engaged in learning and memory processes. The indication of this disorder is the presence of senile plaques and neurofibrillary tangles (NFTs). These senile plaques are extracellular aggregates of amyloid-beta protein produced by the incorrect cleavage of the amyloid precursor protein (APP), and NFTs are intracellular accumulations of abnormal hyperphosphorylated tau proteins. Many hypotheses illustrate these mechanisms, the most accepted of which is the amyloid cascade hypothesis that proposes the abnormal amyloid is processed by beta and gamma secretases and as the main event of AD [107] (Fig. 3).

Although amyloid and tau approaches have been widely adopted and currently are the most studied ones, oxidative stress-based strategies have also been tried, using two different routes: through exogenous antioxidants or by the induction of endogenous antioxidant defenses through the nuclear factor erythroid 2-related factor 2 (Nrf2) [107].

Hymenialdisine belongs to a novel class of CDK inhibitors isolated from Agelasidae, Axinellidae, and Halichondriidae families of marine sponges. The CDK inhibitory efficacy of HD is understood by observing its binding interactions in the CDK2–HD crystal structure. In vivo phosphorylation of particular neuronal proteins by GSK-3 and CDK5 is inhibited by HD. It inhibits the phosphorylation of tau, which is indicative of Alzheimer’s disease. HD could be a lead chemical for analyzing the role of tau hyperphosphorylation in neurodegenerative diseases and specific inhibitors of kinases involved in AD and other degenerative disorders. Several models were used to demonstrate the effects of HD on kinases in vivo. These findings motivated researchers to look into HD as a potential treatment for neurodegenerative diseases [96]. Hymenialdisine also suppresses many pro-inflammatory cytokines (IL-1, IL-2, IL-6, and NO) by inhibition of the NF-kB signaling pathway, which could be useful in the treatment of inflammatory diseases [108].

##### Parkinson’s disease

Parkinson’s disease (PD) is a neurodegenerative disorder caused by the loss of dopaminergic neurons, leading to patients’ motor dysfunctions. Although PD’s etiology is still unclear, the death of dopaminergic neurons during PD progress was revealed to be associated with the abnormal aggregation of synuclein, the elevation of oxidative stress, the dysfunction of mitochondrial functions, and the increase of neuroinflammation. However, current anti-PD therapies could only produce symptom-relieving effects because they could not provide neuroprotective effects and stop or delay dopaminergic neuron degeneration. Marine-derived natural compounds, with their novel chemical structures and unique biological activities, may provide anti-PD neuroprotective effects [109].

Secondary metabolites from marine-derived bacteria represent a rich source for drug development with novel chemical structures and diverse biological activities [110, 111]. NP7 is a marine-derived compound from the *Streptomyces* sp. NP7 is an antioxidant and can pass the blood-brain barrier. NP7 at 5–10M is capable of preventing apoptosis and necrosis induced by H_2_O_2_ in neurons and glial cells [112]. Also, NP7 can inhibit microglial activation and prevent the increased phosphorylation of ERK induced by H_2_O_2_. Therefore, NP7 can act as a neuroprotective agent against oxidative stress in PD [113].

The inhibitory activity of marine-derived compounds piloquinones, isolated from the *Streptomyces* sp., on MAO-B was reported by Takeuchi et al. [114]. Piloquinone A and piloquinone B were isolated from the *Streptomyces* sp. CNQ-027 [115] among which piloquinone (A) is a potent inhibitor of MAO, with an IC_50_ value of 1.21 M for MAO-B and an IC_50_ value of 6.47 M for MAO-A. Simultaneously, piloquinone (B) is only effective against MAO-B, with an IC_50_ value of 14.50 M (63). These results indicated that piloquinone derivatives may be useful lead compounds in the development of MAO-B inhibitors to treat PD.

#### Autoimmune diseases

The autoimmune disease includes rheumatoid arthritis (RA) and other forms of arthritis, type-1 diabetes, heart diseases, irritable bowel syndrome, allergies, asthma, cancer, and many others. Over the past few decades, it was realized that the process of inflammation is virtually the same in different disorders, and a better understanding of inflammation may lead to better treatments for numerous diseases. Inflammation is the activation of the immune system in response to infection, irritation, or injury, with an influx of white blood cells, redness, heat, swelling, pain, and dysfunction of the organs involved. Although these conditions’ pathophysiological basis is not fully understood, reactive oxygen species (ROS) have often been implicated in their pathogenesis. In fact, the antioxidant defense system is compromised in inflammatory diseases, as evidenced by increased oxidative stress markers and decreased protective antioxidant enzymes in patients with rheumatoid arthritis (RA).

##### Secondary metabolites from the Actinomycetes sp. for inflammatory diseases

Cyclomarins are three cyclic heptapeptides (A, B, and C), isolated from the marine bacterium actinomycete, belonging to the *Streptomyces* sp., along the Californian coast. Marine actinomycetes have been exploited as a source of biologically active secondary metabolites with antibacterial and anti-cancer properties [93]. Some molecules have also been reported to be anti-inflammatory, such as cyclomarins and salinamides [116]. Cyclomarin A, constituted of three common and four unusual amino acids, showed potent anti-inflammatory and anti-proliferative activities in in vivo and in vitro assays, managing to inhibit edema pain similar to the drug hydrocortisone [117]. A moderate anti-inflammatory effect has also been reported in cyclomarin C, whose total synthesis was recently experimented and reported [118]. That is why both cyclomarin A and C and their derivatives can act as potent anti-inflammatory therapies naturally.

These five peptides (A, B, C, D, and E) were isolated, like cyclomarin, from marine actinomycetes, belonging to the *Streptomyces* sp., isolated from the surface of the jellyfish *Cassiopeaxamachana*, found in Florida waters [116]. Salinamides A and B are the two primary bicyclic metabolites, with potent topical anti-inflammatory activity and moderate antibiotic activity against gram-positive bacteria, and could be used to treat tissue inflammation and some infections [119].

Spectral and chemical techniques are useful to construct minor metabolites, Salinamides C, D, and E. In salinamide D, a similar structure is observed with isoleucine replaced by valine. Light anti-inflammatory activity is identified in salinamides C and E, thus potentially able to combat inflammatory disease.

#### Different types of cancer

**Fig. 4:**
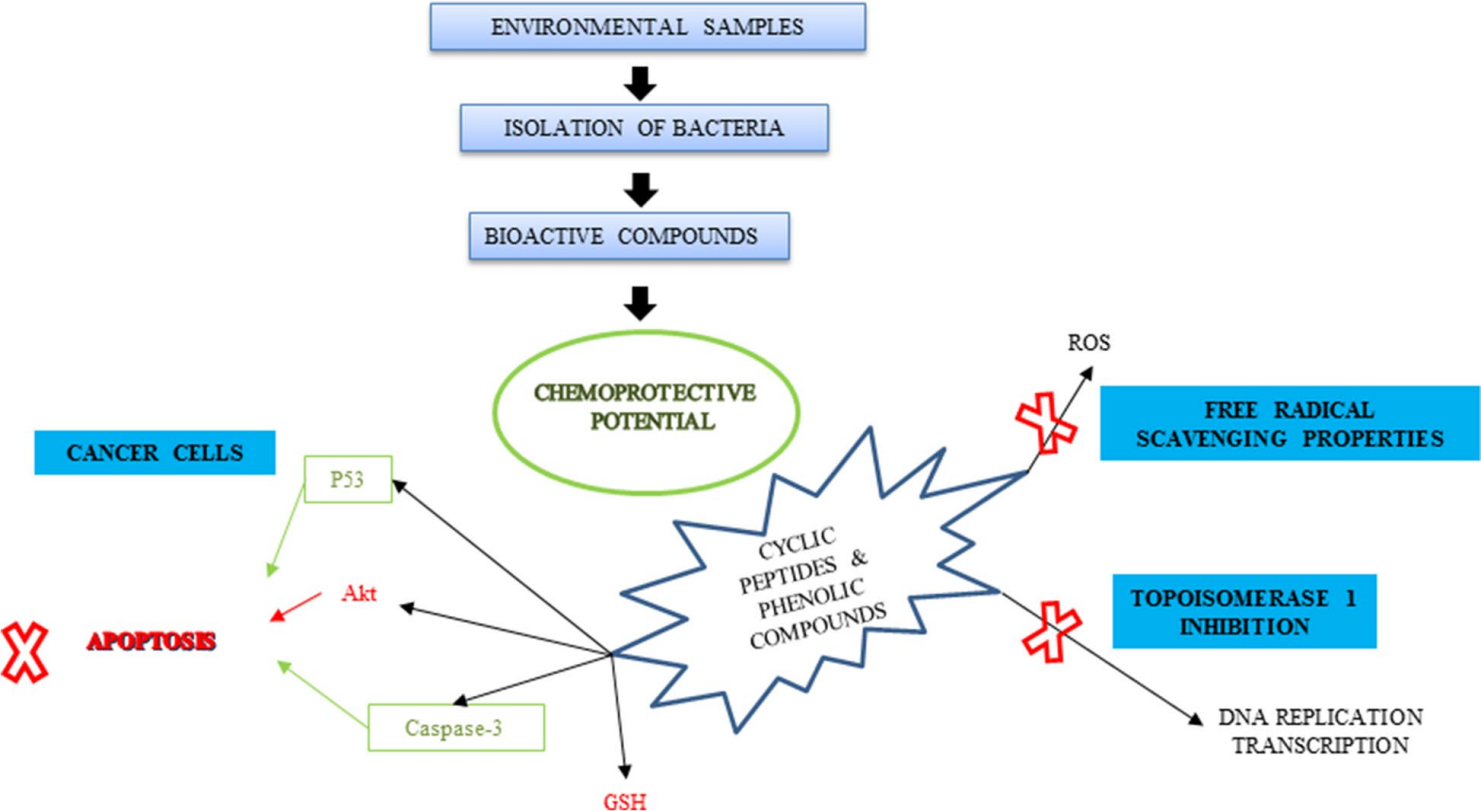
Anti-cancer potential of bioactive compounds from marine source

Chemotherapy is one of the primary therapies against cancer. A significant number of antitumor compounds are natural products or their derivatives, mainly produced by microorganisms. In particular, actinomycetes are the producers of many natural products with different biological activities, including antitumor properties. Several structural classes of antitumor compounds include anthracyclines, enediynes, indolocarbazoles, isoprenoids, macrolides, non-ribosomal peptides, etc. These compounds’ antitumor activity is exerted by inducing apoptosis through DNA cleavage mediated by topoisomerase I or II inhibition, mitochondria permeabilization, and inhibition of key enzymes involved in signal transduction like proteases or cellular metabolism and some cases by inhibiting tumor-induced angiogenesis. Marine organisms have attracted particular attention in the last years for their ability to produce interesting pharmacological lead compounds [120] (Fig. 4).

Many of the antitumor compounds from marine drugs result from marine actinobacteria, and these metabolites show a crucial part in the proof of identity of the pharmaceutical compound. Presently, it seems that there have been only a few studies concentrating on finding therapeutic compounds obtained from marine actinobacteria to be used as anti-cancer agents, as well as anti-infective. Some antitumor compounds from marine sources and their role in different types of cancer are discussed below.

##### Human colon cancer

A high number of type I polyketide-derived compounds with antitumor activity have been isolated from marine actinomycetes. Such is the case of arenicolides, 26-membered polyunsaturated macrolactones, produced by the obligate marine actinomycete *Salinisporaarenicola* strain CNR-005 isolated from a marine sediment sample collected at a depth of 20 m from the coastal water around the island of Guam.

Daryamides also belong to the manumycin family of compounds. They were isolated from *Streptomyces* strain CNQ-085 obtained from marine sediment collected at a depth of 50 m off the San Diego coast, California. Daryamides A to C and (2*E*,4*E*)-7-methylocta-2,4-dienoic acid amide are subjected to cytotoxicity evaluation against the human colon carcinoma cell line HCT-116, showing that daryamide A exhibited significantly more potent cancer cell cytotoxicity, with an IC_50_ of 3.15 μg/mL than daryamides B and C [58].

Marineosins, related to the prodigiosin class of polypyrrole bacterial pigments, are spiroaminal compounds containing two pyrrole functionalities produced by *Streptomyces* strain CNQ- 617 isolated from a marine sediment sample collected offshore of La Jolla, California. Marineosins showed significant inhibition of human colon carcinoma HCT-116 cell line with IC_50_ values of 0.5 μM for marineosin A and 46 micromolar for marineosin B [121].

##### Human cervical cancer

Chalcomycin, a 16-membered macrolide, is produced by the *Streptomyces* sp. M491 isolated from the Qingdao coast (China) [122]. Besides, chalcomycin and the related compound chalcomycin B have been isolated from *Streptomyces* strain B7064 found in mangrove sediments in Hawaii [123]. Chalcomycin is found to inhibit protein synthesis in HeLa human cervix carcinoma cell line [124].

##### Human skin cancer

Human rare macrodiolides composed of dimeric 2-hydroxy-6-alkenyl-benzoic acid lactones with conjugated tetraene-pentahydroxy polyketide chains, produced by the *Marinispora* sp. CNQ-140 was isolated from a sediment sample collected at a depth of 56 m offshore of La Jolla, California. These compounds inhibit cancer cell proliferation with an average LC50 of 0.2-2.7 μM against the NCI’s 60 cancer cell line panel. Marinomycin A showed significant tissue type selectivity being more active against human melanoma cell lines LOX IMVI, M14, SK-MEL-2, SK-MEL-5, UACC-257, and UACC-62 skin cancer [120].

##### Mammary cancer

Manumycin A and chinikomycins A and B (the quinone form of chinikomycin A) were isolated from the *Streptomyces* sp. M045 is derived from the sediment of Jiaozhou Bay in China. Chinikomycins A and B showed moderate antitumor activity. Chinikomycin B showed selective antitumor activity against the mammary cancer cell line MAXF 401NL (IC_50_ of 3.04 μg/mL) [53]. Isolated from the culture broth of *Streptomyces* strain CNH990 isolated from a sediment sample collected at a depth of 20 m at the entrance to the Sea of Cortez, 5 km east of Cabo San Lucas, Mexico [60]. In cytotoxic assays using the human cell line of colon adenocarcinoma HCT-116, marmycin A showed an IC_50_ of 60.5 nM, almost 18 times more potent than marmycin B, which showed an IC_50_ of 1.09 μM. Marmycin A is further evaluated for its *in vitro* cytotoxicity offering a mean IC_50_ value of 0.022 μM against 12 human tumor cell lines (breast, prostate, colon, lung, leukemia).

##### Blood cancer

Nonactin, a cyclic polyether, also known as macrotetrolide, is isolated from cultures of the *Streptomyces* sp. KORDI-3238 from a deep-sea sediment sample collected at Ayu Trough in the western Pacific Ocean [125]. The biosynthesis gene cluster of nonactin has previously isolated and characterized from *S. griseus* DSM40695 [61], revealing that it is synthesized by a non-iteratively acting type II PKS that involves five ketosynthases and lacks the acyl carrier protein. Nonactin is an effective inhibitor against the human K-562 erythroleukemia cell line [126].

Chartreusin is an aromatic glycosylated polyketide, currently in phase II clinical trials [62], that possesses an unusual bislactone synthesized through anthracycline intermediates that might undergo a series of oxidative rearrangements to generate the final bislactone structure. This particular biosynthetic process is unraveled by the isolation of the chartreusin biosynthesis gene cluster from *S. chartreusi*s [127]. Chartreusin shows antitumor activity by binding to DNA, radical-mediated single-strand breaks, and inhibition of topoisomerase II [128].

It possessed significant chemotherapeutic activity against various tumor cell lines such as murine P388 and L1210 leukemia and was identified from Streptomyces sp cultures. FX-58, isolated from marine plant *Salicorniaherbacea* collected in Qingdao, Shandong province, China, showed an inhibitory effect against human tumor cell lines of pro-myelocytic leukemia HL-60, gastric carcinoma BGC-823, and adenocarcinoma MDA-MB-435 with IC_50_ of 6.83, 82.2, and 56.59 μg/mL, respectively.

Altemicidin with a monoterpene-alkaloid skeleton is produced by *Streptomyces sioyaensis* SA-1758 isolated from sea mud collected at Gamo, Miyagi Prefecture, Japan. This compound inhibited the growth of murine lymphoid leukemia L1210 and carcinoma IMC cell lines with IC_50_ values of 0.84 and 0.82 μg/mL, respectively, although it showed high acute toxicity in mice [63].

Streptochlorin is a 3-substituted indole compound with antiangiogenic and anti-cancer activities produced by *Streptomyces* strain 04DH110 isolated from shallow water sediment taken at 1 m depth of Ayajin Bay, on the East Sea of Korea. Streptochlorin exhibited significant *in vitro* growth inhibitory activity against human leukemia K-562 cells with an IC_50_ of 1.05 μg/mL [64].

##### Prostate cancer

Ammosamides are pyrroloiminoquinone compounds produced by *Streptomyces* strain CNR-698 isolated from bottom sediments collected at a depth of 1618 m in the Bahamas Islands. Ammosamide A and B exhibited significant in vitro cytotoxicity against human colon adenocarcinoma HCT-116 cells with an IC_50_ of 320 nM each [129].

##### Hepatic cancer

Caboxamycin is a benzoxazole compound produced by the *Streptomyces* sp. NTK 937 was isolated from an Atlantic Ocean deep sea sediment collected in the Canary Basin. It was tested against different tumor cell lines and showed moderate growth inhibitory activity towards human gastric adenocarcimona AGS, hepatocellular carcinoma Hep G2, and breast carcinoma MCF7 cell lines with GI50 7.5, 7.4, and 7.3 μg/mL, respectively [67].

Compounds of the prodigiosin family, isolated from the *Saccharopolyspora* sp. nov. from sponge Mycale plumose, were collected along the coast of Qingdao, China [130]. The compounds identified as metacycloprodigiosin and undecylprodigiosin [131] exhibited significant cytotoxic activities in vitro, as it is recently described for prodigiosin family of compounds [132], against five cancer cell lines: mouse lymphoma P388, human peripheral blood promyeloblast HL60, lung carcinoma A-549 and SPCA4, and hepatic carcinoma BEL-7402 with IC_50_ values between 0.007 and 7.52 μM for metacycloprodigiosin and 0.013 to 0.11 μM for undecylprodigiosin [130].

#### Marine-derived inhibitors with anticancer activity

A neurotoxic lipoprotein Hoiamide A was isolated from cyanobacterial extracts of the Papua New Guinea cyanobacterium *Symploca* sp. screening inhibitory activity in contrast to 53/Mdm2 interaction (EC_50_ = 4.5 μM) [68, 133]. Niphateolide, a diterpene isolated from the Indonesian sea sponge *Niphates olemda*, is a p53-Hdm2/Mdm2 interaction inhibitor [69]. The marine Actinomycete *Verrucosispora* produces proximicins A, B, and C, which are furan equivalents of netropsin. These support in inducing upregulation of p53 and the cyclin-dependent kinase inhibitor p21 [134]. The *Arthrinium* sp., a marine-derived fungus, was used to isolate hexylitaconic acid. With an IC_50_ of 50 g/mL, it blocked p53/Mdm2 binding [70]. Lissoclinidine B was extracted from *Lissoclinum cf*. *badium*, a cancer-fighting chemical that selectively kills altered cells with wild-type p53 [71].

Anti-mycin analogs from the marine *Streptomyces* sp., N-acetyl-deformylantimycin A (NADA) exhibited an effective way to suppress Hela cells [135]. Himeic acid A is isolated from marine fungus *Aspergillus* sp. exhibited ubiquitin-activating enzyme (E1) inhibitory action at 100 μM [72]. Polyubiquitinated p53 is accumulated in Girolline, a marine sponge isolated from *Cymbastela cantharell* and *Axinella brevistyla* initiating G2/M cell cycle arrest in cancer cells [73]. Leucettamol A isolated from the *Leucetta aff*. *microrhaphis* sea sponge, at 50 μg/ml, inhibits the ubiquitin E2 enzymes Ubc13 and Uev1A by 50% [74].

Dysidiolide is a novel alkyl-hydroxybutenolide diterpene derived from the Bahamas sponge *Dysidea etheria* capable of inhibiting Cdc25 protein phosphatase, causing the G2/M transition of the cell cycle to be delayed by dephosphorylating the p34cdc2/cyclin B complex at Tyr15 and Thr14 residues [75]. Sulfircin, a sesquiterpene sulfate extracted from a marine sponge *Ircinia s*p., had an IC_50_ of 7.8 μM for inhibiting Cdc25 phosphatase [76]. Coscinosulfate is a sesquiterpene sulfate obtained from the new Caledonian sponge *Coscinoderma mathewsi* having significant inhibitory activity towards Cdc25A (IC_50_ = 3μM) [77]. The Fijian sponge *Xestospongia carbonaria* produced halenaquinone, a pentacyclic polykeyide molecule that works as an irreversible inhibitor of recombinant human Cdc25B phosphates (activator of cyclin-dependent kinase Cdc2), which prevents the cell cycle from progressing to the mitotic phase. With an IC_50_ value of 19 μM, this drug displayed an inhibitory effect against the kinase activity of human EGFR [78].

SAD is a mycotoxin that is isolated from *Penicillium oxalicum*. DNA topoisomerase I is inhibited by SAD (MIC = 0.4 μM) and also inhibited the G1 phase of the cell cycle in the GSK-3/-catenin/c-MYC pathway, resulting in considerable cytotoxic action against different cancer cells. SAD slowed the course of the cell cycle in human embryonic palatal mesenchymal cells, preventing them from proliferating [79, 136].

The triterpene Stellettin B was isolated from the sea sponge *Jaspis stellifera*. At a dose of 0.01 μM, this chemical inhibits the development of the glioblastoma cell line SF295 by 50%. Stettettin B's mitotic G1 phase arrest resulted in a decrease in Cdk and an increase in p27 expression. The cleavage of Poly ADP Ribose Polymerase (PARP) and an increase in ROS generation may be linked to apoptosis induction [80].

Phidianidine A is an indole alkaloid isolated from the marine opisthobranch mollusk *Phidiana military* capable of inhibiting CXCL12-induced DNA synthesis, cell migration, and ERK1/2 activation [137, 138]. Fucoidan is a sulfated polysaccharide isolated from brown seaweeds that contains fucose. Fucoidan crude extracts bind CXCL12 and inhibit lung metastasis and tumor growth in 4T1 breast cancer cells [139]. JG6 is a new marine-derived oligosaccharide that has been demonstrated to reduce angiogenesis and tumor metastasis by inhibiting CXCL12/CXCR4 [140].

### Drugs derived from marine sources under clinical trials

#### Phase III

Plitidepsin is a cyclic depsipeptide isolated from a Mediterranean marine tunicate (*Aplidium albicans*) and is structurally linked to didemnins, some of which exhibit antiviral effects [141, 142].

Plitidepsin exhibited high antiviral effectiveness and a favorable therapeutic index in *invitro* models of SARS-CoV-2 infection, outperforming other medicines, including remdesivir, preclinical trials. Notably, plitidepsin has a similar in vitro antiviral impact against the B.1.1.7 variety of SARS-CoV-2, which is known to have multiple mutations altering the viral spike protein, which aids viral entry by interacting with the human ACE2 receptor [143].

Tetrodotoxin (TTX) is a neurotoxin that is primarily present in puffer fish and other marine and terrestrial species. TTX inhibits voltage-gated sodium channels (VGSCs). Some TTX-sensitive VGSCs are extensively expressed by main sensory neurons, and they play a significant role in pain signaling. TTX is now being tested in clinical trials for neuropathic pain caused by chemotherapy and cancer-related pain. Tetrodotoxin has been studied in both preclinical and clinical settings to treat pain caused by neuropathies or cancer and has shown efficacy and a favorable safety profile [144].

#### Phase II

GTS-21 is active in a variety of animal models that are commonly used to study memory and learning. In various in vitro and in vivo investigations, GTS-21 was beneficial in boosting cell survival. GTS-21 is being developed for the treatment of both cognitive dysfunction and neurodegeneration exhibited in Alzheimer’s patients based on its preclinical characteristics. GTS-21 was well tolerated up to 450 mg/day (150 mg t.i.d.) in normal people and showed improvements in cognitive behavior. GTS-21 could be a novel dementia medication, and it should be studied further for its potential therapeutic effects in several disorders affecting cognitive function, including Alzheimer’s disease [145].

Irvalec® (elisidepsin trifluoroacetate, PM02734) is a new marine-derived cyclic peptide from the Kahaladide family in clinical trials with preliminary anticancer efficacy. Previous research has found a link between elisidepsin sensitivity and ErbB3 receptor expression in a panel of NSCLC cell lines [146].

Elisidepsin, in combination with CDDP, TAX, or gemcitabine, could be an effective and viable therapeutic approach that could be tested in several in vivo investigations and give a basis for further development of these combinational treatments in clinical trials in the future. In several cell lines, elisidepsin combined with any of the chemotherapeutic drugs had a synergistic impact. Elisidepsin treatment could influence cells on the lipidic bilayer membrane, which are more likely to possess high numbers of ErbB3 receptors, enhancing the activity of the various medications examined (CDDP, TAX, or gemcitabine). In this regard, cancers with overexpression of ErbB3, such as metastatic breast or lung tumors, could be suitable candidates for these types of combinational trials [147].

#### Phase II

Pseudopterosins and seco-pseudopterosins were isolated from the octocoral Pseudopterogorgia elisabethae of the San Andrés and Providencia islands (southwest Caribbean Sea), and the antimicrobial profile against four pathogenic microorganisms (*Staphylococcus aureus*, *Enterococcus faecalis*, *Pseudomonas aeruginosa*, and *Candida albicans*), as well as a more comprehensive cytotoxic profile against five human cell lines (HeLa, PC-3, HCT116, MCF-7, and BJ) for the compounds PsG, PsP, PsQ, PsS, PsT, PsU, 3-*O*-acetyl-PsU, *seco-*PsJ, *seco-*PsK, and IMNGD were assessed. All of the compounds tested had moderate and non-selective cytotoxic activity against both tumor and normal cell lines, with PsQ and PsG being the most active (GI50 values ranging from 5.8 to 12.0 M). In terms of antimicrobial action, the compounds were shown to have good and selective activity against Gram-positive bacteria, but no activity against Gram-negative bacteria or yeast. PsU, PsQ, PsS, seco-PsK, and PsG were the most active compounds against *S. aureus* (IC_50_ 2.9–4.5 M), and PsG, PsU, and seco-PsK exhibited good activity against *E. faecalis* (IC_50_ 3.1–3.8 M), equivalent to the reference medication vancomycin (4.2 M) [148].

Pseudopterosin H was discovered in the *Pseudopterogorgia elisabethae* marine coral. In vitro screening with the MTT, NBT, and LDH assays, as well as AO/EB fluorescence, was used to examine the therapeutic efficiency of pseudopterosin H on the PC-3 cell line at varying concentrations. Results show that treatment with pseudopterosin H reduces PC-3 cell viability by inducing apoptosis and downregulating the production of intracellular reactive oxygen species. The chemosensitivity of PC-3 cells to pseudopterosin H therapy implies that it could be used as a preventative and therapeutic treatment for metastatic castration-resistant prostate cancer. PsH lowers PC-3 cell viability by causing apoptosis and lowering ROS levels. PsH may directly impact prooxidant enzyme function or indirectly block the pro-inflammatory pathway, NF, resulting in a reduction in ROS. PsH has pharmacological properties that could be beneficial in the treatment of prostate cancer [149].

Bryostatin 1, a marine-derived natural compound, showed procognitive and antidepressant benefits in animals and is currently being tested in human clinical studies for the treatment of Alzheimer’s disease (AD). The effects of bryostatin 1 on the structure and function of hippocampus neurons have been related to its potential to improve learning and memory.

Calvin et al. showed that bryostatin 1 promotes cortical synaptogenesis while lowering dendritic spine density in a protein kinase C (PKC)-dependent manner using a combination of chemical probes and pharmacological inhibitors. Compounds that increase synaptic density while also causing the loss of immature dendritic spines could be a novel pharmaceutical technique for boosting memory by raising the signal-to-noise ratio in the brain [150].

Tissue factor (TF) is a possible target in cervical cancer due to its high expression and link to a poor prognosis. In solid tumors, tisotumab vedotin, a first-in-class experimental antibody–drug combination targeting TF, has shown promising action. Patients with recurrent or metastatic cervical cancer were given tisotumab vedotin 2.0 mg/kg every 3 weeks until their disease progressed, toxicity became unacceptable, or they withdrew their consent. In patients with previously treated recurrent or metastatic cervical cancer, tisotumab vedotin showed a controllable safety profile and promising anticancer efficacy [151].

##### Other drugs derived from marine sources

The hunt for novel chemicals, particularly from marine sources, has piqued the scientific community's interest due to the growing number of diabetic patients and the restricted number of anti-diabetic medications. Marine bioresources have been demonstrated to generate a variety of new scaffolds, several of which have unique structures [152, 153]. Surprisingly, a terpene (Dysidine) isolated from the sponge Dysidea villosa is now being tested in preclinical studies for the treatment of diabetes [154].

Cytarabine (Cytosar-U®, Ara-C, DepoCyt®), an anticancer medication derived from the Caribbean sponge *Tethya crypta*, is used to treat acute myelocytic leukemia and non-Hodgkin’s lymphoma [155, 156]. ET-743 (Yondelis®), derived from the tunicate *Ecteinascidia turbinata*, is approved for the treatment of tissue sarcomas and ovarian cancer, and eribulin (Halaven®), derived from the sponge *Halichondria okadai* [157], is approved for the treatment of metastatic breast cancer and advanced liposarcoma. Marine compounds like ziconotide (Prialt®), obtained from the cone snail *Conus magus* is used to treat severe and chronic pain [158], and vidarabine (Ara-A), isolated from the sponge *Tethya crypta* is used to treat herpes simplex infections [159].

Bioassay-guided fractionation of the EtOAc extract of marine sponges led to the isolation of three polyacetylene metabolites: a new polyacetylene diol, callyspongidiol (**1**), along with two known compounds, siphonodiol (**2**) and 14,15-dihydrosiphonodiol (**3**). Compounds **1**–**3** exhibited antiproliferative activity against HL-60 with IC_50_ values of 6.5, 2.8, and 6.5 μg/ml, respectively. These metabolites induce apoptosis in HL-60 cells [160].

Callyspongidiol and 14,15-dihydrosiphonodiol are polyacetylenediols isolated from marine sponges and are pharmacologically active substances. Callyspongidiol and 14,15-dihydrosiphonodiol activate human DC by phenotypic and functional maturation and altered cytokine production. The results suggested that some polyacetylenediols modulate human DC function in a fashion that favors Th1/Th2 cell polarization or IL-10-producing T cells, and might have implications in tumors or in autoimmune diseases [161].

PP2A inhibition by calyculin-A increased PP2A Y307 phosphorylation without inhibiting oral cancer cells proliferation in both the cell lines. The available data suggested that abnormal, upregulated expression of p-PP2A may promote OSCC proliferation. PP2A plays a major role in various signaling pathways, including those that regulate the cell cycle, cell metabolism, cell migration, and cell survival. Calyculin-A treatment increased AKT (Ser 473) and GSK-3β (Ser9) phosphorylation levels in both the cancer cells, suggesting that this effect occurs via PP2A deactivation. The result suggests that CLA inhibited GSK-3β expression by deactivating PP2A expression [162].

The cone snail *Conus pulicarius* from the Philippines provides a specific habitat for actinomycetes and other bacteria. A phenotypic screen using primary cultures of mouse dorsal root ganglion neurons revealed that one *C. pulicarius* associate, *Streptomyces* sp. CP32, produces a series of natural products that enhance or diminish whole-cell Ca2+ flux. These compounds include thiazoline compounds and a series of new derivatives, pulicatins A–E (6-10) [163].

Arenamides are cyclohexadepsipeptides that are produced via marine bacterial *Salinispora arenicola*. There are three types of these peptides named arenamides A–C. Arenamides A and B block or inhibit the activation of TNF-induce in a dose- and time-dependent manner with IC_50_ values of 3.7 and 1.7 μM, respectively. Furthermore, they are cytotoxic NFkappaB inhibitors and could inhibit the production of nitric oxide (NO) and prostaglandin E2 (PGE2). Also, arenamides A and B show moderate cytotoxic activity against human colon carcinoma cell line HCT-116 [164]. Derivatives of plakortin named gracilioetheres A–C from *Agelas gracilis* were isolated from a bioassay-guided approach from an active extract using *P. falciparum* assay in vitro, highlighting gracilioether B with a IC_50_ value of 1.41 μM and moderate cytoxicity [165].

## Conclusion

In this review, we have identified the derivatives of structurally unique MNPs obtained from marine sources. These MNPs display different potent bioactivities involving not only chemical effects but also pharmaceutical activities, including antibacterial, antiviral, fungicidal, cytotoxic, neurodegenerative, and antimalarial activities because these MNPs derived from marine sources usually contain reactive groups such as -OH, -NH2, and -SH in their chemical structures, and may act as antioxidants. For instance, brown seaweeds contain several bioactive forms, such as omega-3 polyunsaturated fatty acids (PUFAs), polyphenols, fucosterol, and carotenoids at the same time. Marine peptides, marine carotenoids, and marine polyphenols are superior compared to analogous terrestrial resources as they can relieve symptoms and tackle the possible side effects of pharmacological treatment, reducing the risk of complications. The microorganisms associated with the marine environment have great potential as an essential source of structurally exciting molecules. Increasing ocean exploration has brought more marine drugs to the fore. Marine organisms with novel structures and diverse behaviors generate a large number of bioactive compounds. Bioactive compounds that are modified and synthesized from derived leads are directly extracted or isolated from marine species.

Commercial medications remain limited in relieving symptoms and cannot reverse or interrupt the onset or prolong certain diseases’ progression. High cost and adverse side effects of drugs in older adults under treatment involve scientific research falling on natural treatment practices surrounding marine bioactive compounds. Marine-derived compounds have reached ongoing clinical trials against multiple diseases and have become primary drug production sources.

The consideration of marine samples will be an amazing and potential route for identifying new secondary metabolites. It is evident from the study that secondary-metabolite development patterns are highly complex and that molecular studies may enhance drug discovery. Genetic technologies and bioinformatics methods, including metagenomic approaches, genome mining, and heterologous biosynthesis, accelerate the discovery and accessibility of remaining undiscovered MNPs with novel structures and promising marine microorganism bioactivities. It is prominent that implementing multiple techniques and exploration methods could effectively facilitate the exploitation of novel MNPs with various systems. MNPs are well-known sources of secondary metabolites suggesting the potential for pharmaceutical, food, cosmetic, and medical use. Therefore, it is of great economic value and can be used for its industrial and academic needs to its new horizons.

To create new medicines for the future, knowledge about secondary metabolites from marine sources is crucial. They are an essential source of bioactive molecules and inspire drug development by supplying a mixture of several bioactive molecules that can synergize and treat several diseases with biological outcomes.

The scaffolds of terrestrial natural materials are used in more than half of all pharmaceuticals. Despite this, with the introduction of high-throughput screening technology, natural compounds have been overlooked for drug discovery. For successful drug development of complicated structures, several hurdles must be overcome, including the supply problem and target identification. Another complication is that because of variable environmental conditions; the same organism may produce various metabolites at different times. The fact that the bioactive compounds are produced by microbes living in the marine mammal, rather than the invertebrate sea hosts, is a huge obstacle [166]. A sustainable supply of separated and recognized lead compounds can be a challenge if the lead compound is only present in small quantities and/or is difficult to isolate technically [167]. The required quantity for any of the compound’s intended uses (drug, cosmetic, etc.) might range from a few grams for preclinical drug development and safety investigations in various setups to kilograms for clinical studies in various phases [166]. And the quantity of the lead compound can be a significant problem.

Furthermore, obtaining intellectual property (IP) rights for natural products with relevant bioactivities can be difficult, as naturally occurring chemicals are not always patentable in their native form, while simple modifications can be. Because of the complicated structures, the supply problem, and target identification, it is still a challenge for the researchers to translate marine-derived compounds into clinical trials [168]. The effectiveness of marine natural compounds as drug leads depends on advances in technology such as sampling methods, nanoscale NMR for structure characterization, total chemical synthesis, biosynthesis, and genetic engineering. The high level of innovation in the field of marine natural products will lead to successful marine drug discovery and development, giving us reason to believe that marine natural products will form a new wave of drugs that will flood the market and pharmacies in the future.

## Data Availability

Data sharing is not applicable to this article as no datasets were generated or analyzed during the current study.

## Abbreviations

SMs: Secondary metabolites
MNPs: Marine natural products
MRSA: Methicillin-resistant *Staphylococcus aureus*
VRE: Vancomycin-resistant *Enterococcus faecium*
MIC: Minimum inhibitory concentration
DPPH: 2,2-Diphenyl-1-picrylhydrazyl
TNF-alpha: Tumor necrosis factor alpha
IL-1beta: Interleukin-1beta
TLC: Thin-layer chromatography
anti-TB: Anti-tuberculosis
SI: Selectivity index
IC: Inhibitory concentration
ROS: Reactive oxygen species
Nrf2-ARE: Nuclear factor erythroid-2-related factor 2-antioxidant response element
AChE: Acetylcholinesterase
AD: Alzheimer’s disease
PD: Parkinson’s disease
Ab: Amyloid-beta
APP: Amyloid precursor protein
RA: Rheumatoid arthritis
HCT: Human colorectal carcinoma
NCI: National Cancer Institute
PUFAs: Polyunsaturated fatty acids

## References

[1] Bollmann M (2010). World ocean review: living with the oceans.

[2] Subramani R, Aalbersberg W (2013). Culturable rare Actinomycetes: diversity, isolation and marine natural product discovery. Appl Microbiol Biotechnol.

[3] Ramirez-Llodra E, Tyler PA, Baker MC, Bergstad OA, Clark MR, Escobar E, Levin LA, Menot L, Rowden AA, Smith CR (2011). Man and the last great wilderness: human impact on the deep sea. PLoS One.

[4] Newman DJ, Cragg GM (2018) Marine Natural Products with Pharmacological Properties. In Chemical Ecology. CRC Press, pp. 1–25

[5] Atlas RM (1998) Microbial ecology: fundamentals and applications. Pearson Education India

[6] Pinnaka AK, Tanuku NRS (2019) Marine microbial diversity for sustainable development, microbial diversity in ecosystem sustainability and biotechnological applications. Springer, pp 117–158

[7] Mahapatra GP, Raman S, Nayak S, Gouda S, Das G, Patra JK (2020) Metagenomics approaches in discovery and development of new bioactive compounds from marine Actinomycetes. Curr Microbiol 77:645–656 10.1007/s00284-019-01698-5 31069462

[8] Sekurova ON, Schneider O, Zotchev SB (2019). Novel bioactive natural products from bacteria via bioprospecting, genome mining and metabolic engineering. Microb Biotechnol.

[9] Haefner B (2003). Drugs from the deep: marine natural products as drug candidates. Drug Discov Today.

[10] Lahlou M (2013). The success of natural products in drug discovery. Pharmacol Pharm.

[11] Yarza P, Yilmaz P, Pruesse E, Glockner FO, Ludwig W, Schleifer KH, Whitman WB, Euzeby J, Amann R, Rossello-Mora R (2014). Uniting the classification of cultured and uncultured bacteria and archaea using 16S rRNA gene sequences. Nat Rev Microbiol.

[12] Dalmaso GZ, Ferreira D, Vermelho AB (2015). Marine extremophiles: a source of hydrolases for biotechnological applications. Mar Drugs.

[13] Trincone A (2010). Potential biocatalysts originating from sea environments. J Mol Catal B Enzym.

[14] Suleria HAR, Gobe G, Masci P, Osborne SA (2016). Marine bioactive compounds and health promoting perspectives; innovation pathways for drug discovery. Trends Food Sci Technol.

[15] Igarashi Y, Ogura H, Furihata K, Oku N, Indananda C, Thamchaipenet A (2011). Maklamicin, an antibacterial polyketide from an endophytic Micromonospora sp. J Nat Prod.

[16] Igarashi Y, Iida T, Oku N, Watanabe H, Furihata K, Miyanouchi K (2012). Nomimicin, a new spirotetronate-class polyketide from an actinomycete of the genus Actinomadura. J Antibiot (Tokyo).

[17] Niu S, Li S, Chen Y, Tian X, Zhang H, Zhang G, Zhang W, Yang X, Zhang S, Ju J, Zhang C (2011). Lobophorins E and F, new spirotetronate antibiotics from a South China Sea-derived Streptomyces sp. SCSIO 01127. J Antibiot (Tokyo).

[18] Chen C, Wang J, Guo H, Hou W, Yang N, Ren B, Liu M, Dai H, Liu X, Song F, Zhang L (2013). Three antimycobacterial metabolites identified from a marine-derived Streptomyces sp. MS100061. Appl Microbiol Biotechnol.

[19] Rateb ME, Ebel R, Jaspars M (2018). Natural product diversity of actinobacteria in the Atacama Desert. Antonie Van Leeuwenhoek.

[20] Abdelkader MSA, Philippon T, Asenjo JA, Bull AT, Goodfellow M, Ebel R, Jaspars M, Rateb ME (2018). Asenjonamides A-C, antibacterial metabolites isolated from Streptomyces asenjonii strain KNN 42.f from an extreme-hyper arid Atacama Desert soil. J Antibiot (Tokyo).

[21] Hou J, Liu P, Qu H, Fu P, Wang Y, Wang Z, Li Y, Teng X, Zhu W (2012). Gilvocarcin HE: a new polyketide glycoside from Streptomyces sp. J Antibiot (Tokyo).

[22] Lü Y, Shao M, Wang Y, Qian S, Wang M, Wang Y, Li X, Bao Y, Deng C, Yue C, Liu D, Liu N, Liu M, Huang Y, Chen Z, Hu Y (2017). Zunyimycins B and C, New chloroanthrabenzoxocinones antibiotics against methicillin-resistant Staphylococcus aureus and Enterococci from Streptomyces sp. FJS31-2. Molecules (Basel, Switzerland).

[23] Qin Z, Munnoch JT, Devine R, Holmes NA, Seipke RF, Wilkinson KA, Wilkinson B, Hutchings MI (2017). Formicamycins, antibacterial polyketides produced by Streptomyces formicae isolated from African Tetraponera plant-ants. Chem Sci.

[24] Cruz JC, Maffioli SI, Bernasconi A, Brunati C, Gaspari E, Sosio M, Wellington E, Donadio S (2017). Allocyclinones, hyperchlorinated angucyclinones from Actinoallomurus. J Antibiot (Tokyo).

[25] Rathod BB, Korasapati R, Sripadi P, Reddy Shetty P (2018). Novel actinomycin group compound from newly isolated Streptomyces sp. RAB12: isolation, characterization, and evaluation of antimicrobial potential. Appl Microbiol Biotechnol.

[26] Cheng C, Othman EM, Reimer A, Grüne M, Kozjak-Pavlovic V, Stopper H, Hentschel U, Abdelmohsen UR (2016). Ageloline A, new antioxidant and antichlamydial quinolone from the marine sponge-derived bacterium Streptomyces sp. SBT345. Tetrahedron Lett.

[27] Loureiro DRP, Soares JX, Costa JC, Magalhaes AF, Azevedo CMG, Pinto MMM, Afonso CMM (2019). Structures, activities and drug-likeness of anti-infective xanthone derivatives isolated from the marine environment: a review. Molecules.

[28] Liu LL, Xu Y, Han Z, Li YX, Lu L, Lai PY, Zhong JL, Guo XR, Zhang XX, Qian PY (2012). Four new antibacterial xanthones from the marine-derived actinomycetes Streptomyces caelestis. Mar Drugs.

[29] Mahajan G, Thomas B, Parab R, Patel ZE, Kuldharan S, Yemparala V, Mishra PD, Ranadive P, D’Souza L, Pari K, Sivaramkrishnan H (2013). In vitro and in vivo activities of antibiotic PM181104. Antimicrob Agents Chemother.

[30] Wu Z, Li S, Li J, Chen Y, Saurav K, Zhang Q, Zhang H, Zhang W, Zhang W, Zhang S, Zhang C (2013). Antibacterial and cytotoxic new napyradiomycins from the marine-derived Streptomyces sp. SCSIO 10428. Mar Drugs.

[31] Shin B, Kim BY, Cho E, Oh KB, Shin J, Goodfellow M, Oh DC (2016). Actinomadurol, an antibacterial norditerpenoid from a rare actinomycete, Actinomadura sp. KC 191. J Nat Prod.

[32] Manam RR, Teisan S, White DJ, Nicholson B, Grodberg J, Neuteboom ST, Lam KS, Mosca DA, Lloyd GK, Potts BC (2005). Lajollamycin, a nitro-tetraene spiro-beta-lactone-gamma-lactam antibiotic from the marine actinomycete Streptomyces nodosus. J Nat Prod.

[33] Um S, Choi TJ, Kim H, Kim BY, Kim SH, Lee SK, Oh KB, Shin J, Oh DC (2013). Ohmyungsamycins A and B: cytotoxic and antimicrobial cyclic peptides produced by Streptomyces sp. from a volcanic island. J Org Chem.

[34] Sun P, Maloney KN, Nam SJ, Haste NM, Raju R, Aalbersberg W, Jensen PR, Nizet V, Hensler ME, Fenical W (2011). Fijimycins A-C, three antibacterial etamycin-class depsipeptides from a marine-derived Streptomyces sp. Bioorg Med Chem.

[35] Shazleen AA (2017). Dissertation. Discovery of novel rare actinobacteria isolated from mangrove environments in the east coast of Peninsular Malaysia.

[36] Veena S, Swetha D, Karthik L, Bhaskara Rao K (2016). Assessment of anti-typhoid and antioxidant activity of marine actinobacteria isolated from Chennai marine sediments. Der Pharm Lett.

[37] Vijayakumar R, Muthukumar C, Thajuddin N, Panneerselvam A, Saravanamuthu R (2007) Studies on the diversity of actinomycetes in the Palk Strait region of Bay of Bengal, India. Actinomycetologica 21(2):0712050027

[38] Avilala J, Kumar AP, Viswanath B, Gopal DVRS, Narasimha G (2018). Antiviral and larvicidal properties of novel bioactive compounds produced from marine actinomycetes. Russ J Mar Biol.

[39] Saha S, Dhanasekaran D, Shanmugapriya S, Latha S (2013). Nocardiopsis sp. SD5: a potent feather degrading rare actinobacterium isolated from feather waste in Tamil Nadu, India. J Basic Microbiol.

[40] Kirst HA (2010). The spinosyn family of insecticides: realizing the potential of natural products research. J Antibiot.

[41] Dhanasekaran D, Sakthi V, Thajuddin N, Panneerselvam A (2010) Preliminary evaluation of anopheles mosquito larvicidal efficacy of mangrove actinobacteria. Int J Appl Biol Pharm 1(2):374–81

[42] Vo TS, Ngo DH, Kang KH, Jung WK, Kim SK (2015). The beneficial properties of marine polysaccharides in alleviation of allergic responses. Mol Nutr Food Res.

[43] Cardoso ML, Xavier CA, Bezerra MB, Paiva AO, Carvalho MF, Benevides NM, Rocha FA, Leite EL (2010). Assessment of zymosan-induced leukocyte influx in a rat model using sulfated polysaccharides. Planta Med.

[44] Matsui MS, Muizzuddin N, Arad S, Marenus K (2003). Sulfated polysaccharides from red microalgae have antiinflammatory properties in vitro and in vivo. Appl Biochem Biotechnol.

[45] Medeiros VP, Queiroz KCS, Cardoso ML, Monteiro GRG, Oliveira FW, Chavante SF, Guimaraes LA, Rocha HAO, Leite EL (2008). Sulfated galactofucan from Lobophora variegata: anticoagulant and anti-inflammatory properties. Biochem (Moscow).

[46] De Jesus Raposo MS, De Morais AM, De Morais RM (2015). Marine polysaccharides from algae with potential biomedical applications. Mar Drugs.

[47] Cheung RC, Wong JH, Pan W, Chan YS, Yin C, Dan X, Ng TB (2015). Marine lectins and their medicinal applications. Appl Microbiol Biotechnol.

[48] Da Conceicao Rivanor RL, Chaves HV, Do Val DR, De Freitas AR, Lemos JC, Rodrigues JA, Pereira KM, De Araujo IW, Bezerra MM, Benevides NM (2014). A lectin from the green seaweed Caulerpa cupressoides reduces mechanical hyper-nociception and inflammation in the rat temporomandibular joint during zymosan-induced arthritis. Int Immunopharmacol.

[49] Sepcic K, Marcel V, Klaebe A, Turk T, Suput D, Fournier D (1998). Inhibition of acetylcholinesterase by an alkylpyridinium polymer from the marine sponge, Reniera sarai. Biochim Biophys Acta.

[50] Tan L, Guo S, Ma F, Chang, Gómez-Betancur I (2018). In vitro inhibition of acetylcholinesterase, alphaglucosidase, and xanthine oxidase by bacteria extracts from coral reef in Hainan, South China Sea. J Mar Sci Eng.

[51] Mitchell SS, Nicholson B, Teisan S, Lam KS, Potts BC (2004). Aureoverticillactam, a novel 22-atom macrocyclic lactam from the marine actinomycete Streptomyces aureoverticillatus. J Nat Prod.

[52] Stritzke K, Schulz S, Laatsch H, Helmke E, Beil W (2004). Novel caprolactones from a marine streptomycete. J Nat Prod.

[53] Li F, Maskey RP, Qin S, Sattler I, Fiebig H, Maier A, Zeeck A, Laatsch H (2005). Chinikomycins A and B: isolation, structure elucidation, and biological activity of novel antibiotics from a marine Streptomyces sp. isolate M045. J Nat Prod.

[54] Malet-Cascon L, Romero F, Espliego-Vazquez F, Gravalos D, Fernandez-Puentes JL (2003). IB-00208, a new cytotoxic polycyclic xanthone produced by a marine-derived Actinomadura. I. Isolation of the strain, taxonomy and biological activities. J Antibiot (Tokyo).

[55] Beer LL, Moore BS (2007). Biosynthetic convergence of salinosporamides A and B in the marine actinomycete Salinispora tropica. Org Lett.

[56] Ganesan S, Velsamy G, Sivasudha T, Manoharan N (2013). MALDI-TOF mass spectrum profiling, antibacterial and anticancer activity of marine Streptomyces fradiae BDMS1. World J Pharm Pharm Sci.

[57] Leet JE, Schroeder DR, Golik J, Matson JA, Doyle TW, Lam KS, Hill SE, Lee MS, Whitney JL, Krishnan BS (1996). Himastatin, a new antitumor antibiotic from Streptomyces hygroscopicus. III. Structural elucidation. J Antibiot (Tokyo).

[58] Asolkar RN, Jensen PR, Kauffman CA, Fenical W (2006). Daryamides A-C, weakly cytotoxic polyketides from a marine-derived actinomycete of the genus Streptomyces strain CNQ-085. J Nat Prod.

[59] Kim SK, Hoang VL, Kim MM (2006). Bioactive compounds derived from marine bacteria: anti-cancer activity. J Mar Biosci Biotechnol.

[60] Martin GD, Tan LT, Jensen PR, Dimayuga RE, Fairchild CR, Raventos-Suarez C, Fenical W (2007). Marmycins A and B, cytotoxic pentacyclic C-glycosides from a marine sediment-derived actinomycete related to the genus Streptomyces. J Nat Prod.

[61] Smith WC, Xiang L, Shen B (2000). Genetic localization and molecular characterization of the nonS gene required for macrotetrolide biosynthesis in Streptomyces griseus DSM40695. Antimicrob Agents Chemother.

[62] Butler MS (2008). Natural products to drugs: natural product-derived compounds in clinical trials. Nat Prod Rep.

[63] Takahashi A, Kurasawa S, Ikeda D, Okami Y, Takeuchi T (1989). Altemicidin, a new acaricidal and antitumor substance. J Antibiot.

[64] Shin HJ, Jeong HS, Lee HS, Park SK, Kim HM, Kwon HJ (2007). Isolation and structure determination of streptochlorin, an antiproliferative agent from a marine-derived Streptomyces sp. 04DH110. J Microbiol Biotechnol.

[65] Boonlarppradab C, Kauffman CA, Jensen PR, Fenical W (2008). Marineosins A and B, cytotoxic spiroaminals from a marine-derived actinomycete. Org Lett.

[66] Pan E, Oswald NW, Legako AG, Life JM, Posner BA, Macmillan JB (2013). Precursor-directed generation of amidine containing ammosamide analogs: ammosamides E-P. Chem Sci.

[67] Losada AA, Cano-Prieto C, Garcia-Salcedo R, Brana AF, Mendez C, Salas JA, Olano C (2017). Caboxamycin biosynthesis pathway and identification of novel benzoxazoles produced by cross-talk in Streptomyces sp. NTK 937. Microb Biotechnol.

[68] Malloy KL, Choi H, Fiorilla C, Valeriote FA, Matainaho T, Gerwick WH (2012). Hoiamide D, a marine cyanobacteria-derived inhibitor of p53/MDM2 interaction. Bioorg Med Chem Lett.

[69] Kato H, Nehira T, Matsuo K, Kawabata T, Kobashigawa Y, Morioka H, Losung F, Mangindaan RE, De Voogd NJ, Yokosawa H (2015). Niphateolide A: isolation from the marine sponge Niphates olemda and determination of its absolute configuration by an ECD analysis. Tetrahedron.

[70] Tsukamoto S, Yoshida T, Hosono H, Ohta T, Yokosawa H (2006). Hexylitaconic acid: a new inhibitor of p53–HDM2 interaction isolated from a marine-derived fungus, Arthrinium sp. Bioorg Med Chem Lett.

[71] Clement JA, Kitagaki J, Yang Y, Saucedo CJ, O’Keefe BR, Weissman AM, McKee TC, McMahon JB (2008). Discovery of new pyridoacridine alkaloids from Lissoclinum cf. badium that inhibit the ubiquitin ligase activity of Hdm2 and stabilize p53. Bioorg Med Chem.

[72] Tsukamoto S, Hirota H, Imachi M, Fujimuro M, Onuki H, Ohta T, Yokosawa H (2005). Himeic acid A: a new ubiquitin-activating enzyme inhibitor isolated from a marine-derived fungus, Aspergillus sp. Bioorg Med Chem Lett.

[73] Tsukamoto S, Yamashita K, Tane K, Kizu R, Ohta T, Matsunaga S, Fusetani N, Kawahara H, Yokosawa H, Bulletin P (2004). Girolline, an antitumor compound isolated from a sponge, induces G2/M cell cycle arrest and accumulation of polyubiquitinated p53. Biol Pharm Bul.

[74] Tsukamoto S, Takeuchi T, Rotinsulu H, Mangindaan RE, Van Soest RW, Ukai K, Kobayashi H, Namikoshi M, Ohta T, Yokosawa H (2008). Leucettamol A: a new inhibitor of Ubc13-Uev1A interaction isolated from a marine sponge, Leucetta aff. Microrhaphis Bioorg Med Chem Lett.

[75] Gunasekera SP, McCarthy PJ, Kelly-Borges M, Lobkovsky E, Clardy J (1996). Dysidiolide: a novel protein phosphatase inhibitor from the Caribbean sponge Dysidea etheria de Laubenfels. J Am Chem Soc.

[76] Nagle DG, Zhou YD, Mora FD, Mohammed KA, Kim YP (2004). Mechanism targeted discovery of antitumor marine natural products. Curr Med Chem.

[77] Loukaci S, Le Saout I, Samadi M, Leclerc S, Damiens E, Meijer L, Debitus C, Guyot M (2001). Coscinosulfate, a CDC25 phosphatase inhibitor from the sponge Coscinoderma mathewsi. Bioorg Med Chem.

[78] Skropeta D, Pastro N, Zivanovic A (2011). Kinase inhibitors from marine sponges Mar. Drugs.

[79] Cherigo L, Lopez D, Martinez-Luis S (2015). Marine natural products as breast cancer resistance protein inhibitors. Mar Drugs.

[80] Wang R, Zhang Q, Peng X, Zhou C, Zhong Y, Chen X, Qiu Y, Jin M, Gong M, Kong D (2016). Stellettin B induces G1 arrest, apoptosis and autophagy in human non-small cell lung cancer A549 cells via blocking PI3K/Akt/mTOR pathway. Sci Rep.

[81] Bister B, Bischoff, Strobele M, Riedlinger J, Reicke A, Wolter F, Bull AT, Zahner H, Fiedler HP, Sussmuth RD (2004). Abyssomicin C-A polycyclic antibiotic from a marine Verrucosispora strain as an inhibitor of the p-aminobenzoic acid/tetrahydrofolate biosynthesis pathway. Angew Chem Int Ed Engl.

[82] Bruntner C, Binder T, Pathom-aree W, Goodfellow M, Bull AT, Potterat O, Puder C, Horer S, Schmid A, Bolek W, Wagner K, Mihm G, Fiedler HP (2005). Frigocyclinone, a novel angucyclinone antibiotic produced by a Streptomyces griseus strain from Antarctica. J Antibiot (Tokyo).

[83] Lu Y, Dong X, Liu S, Bie X (2009). Characterization and identification of a novel marine Streptomyces sp. produced antibacterial substance. Mar Biotechnol (NY).

[84] Maskey RP, Helmke E, Kayser O, Fiebig HH, Maier A, Busche A, Laatsch H (2004). Anti-cancer and antibacterial trioxacarcins with high anti-malaria activity from a marine Streptomycete and their absolute stereochemistry. J Antibiot.

[85] Flora DO, Adeyemi AI, George WP (2015). Himalomycin A and cycloheximide-producing marine actinomycete from Lagos Lagoon soil sediment. J Coast Life Med.

[86] Phan LY, Jian T, Chen Z, Qiu YL, Wang Z, Beach T, Polemeropoulos A, Or YS (2004). Synthesis and antibacterial activity of a novel class of 4’-substituted 16-membered ring macrolides derived from tylosin. J Med Chem.

[87] Igarashi M, Sawa R, Yamasaki M, Hayashi C, Umekita M, Hatano M, Fujiwara T, Mizumoto K, Nomoto A (2017). Kribellosides, novel RNA 5’-triphosphatase inhibitors from the rare actinomycete Kribbella sp. MI481-42F6. J Antibiot (Tokyo).

[88] Brana AF, Sarmiento-Vizcaino A, Osset M, Perez-Victoria I, Martin J, De Pedro N, De la Cruz M, Diaz C, Vicente F, Reyes F, Garcia LA, Blanco G (2017). Lobophorin K, a new natural product with cytotoxic activity produced by Streptomyces sp. M-207 associated with the deep-sea coral Lophelia pertusa. Mar Drugs.

[89] Moon K, Chung B, Shin Y, Lee SK, Oh KB, Shin J, Oh DC (2015) Discovery of new bioactive secondary metabolites from bacteria in extreme habitats. Planta Med 81(11):PT24

[90] Rao M, Wei W, Ge M, Chen D, Sheng X (2013). A new antibacterial lipopeptide found by UPLC-MS from an actinomycete Streptomyces sp. HCCB10043. Nat Prod Res.

[91] Ramalingam V, Varunkumar K, Ravikumar V, Rajaram R (2018). p53 mediated transcriptional regulation of long non-coding RNA by 1-hydroxy-1-norresistomycin triggers intrinsic apoptosis in adenocarcinoma lung cancer. Chem Biol Interact.

[92] Yu Y, Wu J, Lei F, Chen L, Wan W, Hai L, Guan M, Wu Y (2013). Design, synthesis and anticancer activity evaluation of diazepinomicin derivatives. Lett Drug Des Disc.

[93] Jensen PR, Williams PG, Oh DC, Zeigler L, Fenical W (2007). Species-specific secondary metabolite production in marine actinomycetes of the genus Salinispora. Appl Environ Microbiol.

[94] Itoh T, Kinoshita M, Aoki S, Kobayashi M (2003). Komodoquinone A, a novel neuritogenic anthracycline, from marine Streptomyces sp. KS3. J Nat Prod.

[95] Oja T, San Martin Galindo P, Taguchi T, Manner S, Vuorela PM, Ichinose K, Metsa-Ketela M, Fallarero A (2015). Effective antibiofilm polyketides against Staphylococcus aureus from the pyranonaphthoquinone biosynthetic pathways of Streptomyces species. Antimicrob Agents Chemother.

[96] Meijer L, Thunnissen AM, White A, Garnier M, Nikolic M, Tsai L, Walter J, Cleverley K, Salinas P, Wu Y, Biernat J (2000). Inhibition of cyclin-dependent kinases, GSK-3β and CK1 by hymenialdisine, a marine sponge constituent. Chem Biol.

[97] Barry CE, Slayden RA, Sampson AE, Lee RE (2000). Use of genomics and combinatorial chemistry in the development of new antimycobacterial drugs. Biochem Pharm.

[98] Copp BR (2003). Antimycobacterial natural products. Nat Prod Rep.

[99] Tiberi S, Munoz-Torrico M, Duarte R, Dalcolmo M, D’Ambrosio L, Migliori GB (2018). New drugs and perspectives for new anti-tuberculosis regimens. Pulmonology.

[100] Patridge E, Gareiss P, Kinch MS, Hoyer D (2016). An analysis of FDA-approved drugs: natural products and their derivatives. Drug Discov Today.

[101] Leiros M, Alonso E, Rateb ME, Ebel R, Jaspars M, Alfonso A, Botana LM (2015). The Streptomyces metabolite anhydroexfoliamycin ameliorates hallmarks of Alzheimer’s disease in vitro and in vivo. Neuroscience.

[102] Adsersen A, Gauguin B, Gudiksen L, Jager AK (2006). Screening of plants used in Danish folk medicine to treat memory dysfunction for acetylcholinesterase inhibitory activity. J Ethnopharmacol.

[103] Dohi S, Terasaki M, Makino M (2009). Acetylcholinesterase inhibitory activity and chemical composition of commercial essential oils. J Agric Food Chem.

[104] Chang CJ, Floss HG, Soong P, Chang CT (1975). Identity of the antitumor antibiotic litmomycin with granaticin A. J Antibiot (Tokyo).

[105] Elson AL, Box SJ, Gilpin ML (1988). New quinone antibiotics of the granaticin type, isolated from Streptomyces lateritius. I. Production, isolation and properties. J Antibiot (Tokyo).

[106] Almasi F, Mohammadipanah F, Adhami HR, Hamedi J (2018). Introduction of marine-derived Streptomyces sp. UTMC 1334 as a source of pyrrole derivatives with anti-acetylcholinesterase activity. J Appl Microbiol.

[107] Karran E, Mercken M, De Strooper B (2011). The amyloid cascade hypothesis for Alzheimer’s disease: an appraisal for the development of therapeutics. Nat Rev Drug Discov.

[108] Sharma V, Lansdell TA, Jin G, Tepe JJ (2004). Inhibition of cytokine production by hymenialdisine derivatives. J Med Chem.

[109] Huang C, Zhang Z, Cui W (2019). Marine-derived natural compounds for the treatment of Parkinson’s disease. Mar Drugs.

[110] Nikapitiya C (2012). Bioactive secondary metabolites from marine microbes for drug discovery. Adv Food Nutr Res.

[111] Monciardini P, Iorio M, Maffioli S, Sosio M, Donadio S (2014). Discovering new bioactive molecules from microbial sources. Microb Biotechnol.

[112] Koppula S, Kumar H, More SV, Kim BW, Kim IS, Choi DK (2012). Recent advances on the neuroprotective potential of antioxidants in experimental models of Parkinson’s disease. Int J Mol Sci.

[113] Mena MA, Casarejos MJ, Solano R, Rodriguez-Navarro JA, Gomez A, Rodal I, Medina M, De Yebenes JG (2009). NP7 protects from cell death induced by oxidative stress in neuronal and glial midbrain cultures from parkin null mice. FEBS Lett.

[114] Takeuchi T, Ogawa K, Iinuma H, Suda H, Ukita K (1973). Monoamine oxidase inhibitors isolated from fermented broths. J Antibiot (Tokyo).

[115] Lee HW, Choi H, Nam SJ, Fenical W, Kim H (2017). Potent inhibition of monoamine oxidase B by a piloquinone from marine-derived Streptomyces sp. CNQ-027. J Microbiol Biotechnol.

[116] Moore BS, Trischman JA, Seng D, Kho D, Jensen PR, Fenical W (1999). Salinamides, antiinflammatory depsipeptides from a marine streptomycete. J Org Chem.

[117] Renner MK, Shen YC, Cheng XC, Jensen PR, Frankmoelle W, Kauffman CA, Fenical W, Lobkovsky E, Clardy J (1999). Cyclomarins A−C, new antiinflammatory cyclic peptides produced by a marine bacterium (Streptomyces sp.). J Am Chem Soc.

[118] Wen SJ, Hu TS, Yao ZJ (2005). Macrocyclization studies and total synthesis of cyclomarin C, an anti-inflammatory marine cyclopeptide. Tetrahedron.

[119] Pietra F (1997). Secondary metabolites from marine microorganisms: bacteria, protozoa, algae and fungi. Achievements and prospects. Nat Prod Rep.

[120] Gomathi A, Gothandam KM (2016) Ocean dwelling actinobacteria as source of antitumor compounds. Braz Arch Biol Technol 59

[121] Kwon HC, Kauffman CA, Jensen PR, Fenical W (2006). Marinomycins A−D, antitumor-antibiotics of a new structure class from a marine actinomycete of the recently discovered genus “Marinispora”. J Am Chem Soc.

[122] Wu SJ, Fotso S, Li F, Qin S, Laatsch H (2007). Amorphane sesquiterpenes from a marine Streptomyces sp. J Nat Prod.

[123] Asolkar RN, Maskey RP, Helmke E, Laatsch H (2002). Chalcomycin B, a new macrolide antibiotic from the marine isolate Streptomyces sp. B7064. J Antibiot (Tokyo).

[124] Gupta RS, Murray W, Gupta R (1988). Cross resistance pattern towards anticancer drugs of a human carcinoma multidrug-resistant cell line. Br J Cancer.

[125] Jeong SY, Shin HJ, Kim TS, Lee HS, Park SK, Kim HM (2006). Streptokordin, a new cytotoxic compound of the methylpyridine class from a marine-derived Streptomyces sp. KORDI-3238. J Antibiot (Tokyo).

[126] Borrel MN, Pereira E, Fiallo M, Garnier-Suillerot A (1994). Mobile ionophores are a novel class of P-glycoprotein inhibitors. The effects of ionophores on 4’-O-tetrahydropyranyl-adriamycin incorporation in K562 drug-resistant cells. Eur J Biochem.

[127] Xu Z, Jakobi K, Welzel K, Hertweck C (2005). Biosynthesis of the antitumor agent chartreusin involves the oxidative rearrangement of an anthracyclic polyketide. Chem Biol.

[128] Lorico A, Long BH (1993). Biochemical characterisation of elsamicin and other coumarin-related antitumour agents as potent inhibitors of human topoisomerase II. Eur J Can.

[129] Hughes CC, MacMillan JB, Gaudencio SP, Jensen PR, Fenical W (2009). The ammosamides: structures of cell cycle modulators from a marine-derived Streptomyces species. Angew Chem Int Ed Engl.

[130] Liu R, Cui CB, Duan L, Gu QQ, Zhu WM (2005). Potent in vitro anticancer activity of metacycloprodigiosin and undecylprodigiosin from a sponge-derived actinomycete Saccharopolyspora sp. nov. Arch Pharm Res.

[131] Wasserman H, Keith D, Rodgers G (1976). The structure of metacycloprodigiosin. Tetrahedron.

[132] Perez-Tomas R, Montaner B, Llagostera E, Soto-Cerrato V (2003). The prodigiosins, proapoptotic drugs with anticancer properties. Biochem Pharmacol.

[133] Mi Y, Zhang J, He S, Yan X (2017). New peptides isolated from marine cyanobacteria, an overview over the past decade. Mar Drugs.

[134] Schneider K, Keller S, Wolter FE, Röglin L, Beil W, Seitz O, Nicholson G, Bruntner C, Riedlinger J, Fiedler HP (2008). Proximicins A, B, and C—antitumor furan analogues of netropsin from the marine actinomycete Verrucosispora induce upregulation of p53 and the cyclin kinase inhibitor p21. Angew Chem Int Ed Engl.

[135] Zhang W, Che Q, Tan H, Qi X, Li J, Li D, Gu Q, Zhu T, Liu M (2017). Marine Streptomyces sp. derived antimycin analogues suppress HeLa cells via depletion HPV E6/E7 mediated by ROS-dependent ubiquitin–proteasome system. Sci Rep.

[136] Zhang JY, Tao LY, Liang YJ, Yan YY, Dai CL, Xia XK, She ZG, Lin YC, Fu LW (2009). Secalonic acid D induced leukemia cell apoptosis and cell cycle arrest of G1 with involvement of GSK-3β/β-catenin/c-Myc pathway. Cell Cycle.

[137] Barbieri F, Thellung S, Würth R, Gatto F, Corsaro A, Villa V, Nizzari M, Albertelli M, Ferone D, Florio T (2014). Emerging targets in pituitary adenomas: role of the CXCL12/CXCR4-R7 system. Int J Endocrinol.

[138] Vitale RM, Gatti M, Carbone M, Barbieri F, Felicità V, Gavagnin M, Florio T, Amodeo P (2013). Minimalist hybrid ligand/receptor-based pharmacophore model for CXCR4 applied to a small-library of marine natural products led to the identification of phidianidine a as a new CXCR4 ligand exhibiting antagonist activity. Chem Biol.

[139] Kwak JY (2014). Fucoidan as a marine anticancer agent in preclinical development. Mar Drugs.

[140] Sharma A, Nandi S (2020). Abnormal signal transduction via over-expression of Pim-1 regulated senescence, cell cycle, apoptosis and metastatic invasion: novel anticancer targets and their potent inhibitors from marine sources. Curr Signal Trans Ther.

[141] Rinehart KL, Gloer JB, Hughes RG, Renis HE, McGovren JP, Swynenberg EB, Stringfellow DA, Kuentzel SL, Li LH (1981). Didemnins: antiviral and antitumor depsipeptides from a Caribbean tunicate. Science.

[142] Canonico PG, Pannier WL, Huggins JW, Rienehart KL (1982). Inhibition of RNA viruses in vitro and in Rift Valley fever-infected mice by didemnins A and B. Antimicrob Agents Chemother.

[143] Reuschl AK, Thorne LG, Zuliani-Alvarez L, Bouhaddou M, Obernier K, Soucheray M, Turner J, Fabius JM, Nguyen GT, Swaney DL, Rosales R (2021). Host-directed therapies against early-lineage SARS-CoV-2 retain efficacy against B. 1.1. 7 variant. BioRxiv.

[144] González-Cano R, Ruiz-Cantero MC, Santos-Caballero M, Gómez-Navas C, Tejada MA, Nieto FR (2021). Tetrodotoxin, a potential drug for neuropathic and cancer pain relief?. Toxins.

[145] Kitagawa H, Takenouchi T, Azuma R, Wesnes KA, Kramer WG, Clody DE, Burnett AL (2003). Safety, pharmacokinetics, and effects on cognitive function of multiple doses of GTS-21 in healthy, male volunteers. Neuropsychopharmacology.

[146] Teixidó C, Arguelaguet E, Pons B, Aracil M, Jimeno J, Somoza R, Marés R, Ramón y Cajal S, Hernández-Losa J (2012). ErbB3 expression predicts sensitivity to elisidepsin treatment: in vitro synergism with cisplatin, paclitaxel and gemcitabine in lung, breast and colon cancer cell lines. Int J Oncol.

[147] Xue C, Liang F, Mahmood R, Vuolo M, Wyckoff J, Qian H, Tsai KL, Kim M, Locker J, Zhang ZY, Segall JE (2006). ErbB3-dependent motility and intravasation in breast cancer metastasis. Cancer Res.

[148] Correa H, Aristizabal F, Duque C, Kerr R (2011). Cytotoxic and antimicrobial activity of pseudopterosins and seco-pseudopterosins isolated from the octocoral Pseudopterogorgia elisabethae of San Andrés and Providencia Islands (Southwest Caribbean Sea). Mar Drugs.

[149] Bowers Z, Caraballo D, Bentley A (2021). Therapeutic potential of pseudopterosin H on a prostate cancer cell line. J Cancer Prev Curr Res.

[150] Ly C, Shimizu AJ, Vargas MV, Duim WC, Wender PA, Olson DE (2020). Bryostatin 1 promotes synaptogenesis and reduces dendritic spine density in cortical cultures through a PKC-dependent mechanism. ACS Chem Neurosci.

[151] Hong DS, Concin N, Vergote I, De Bono JS, Slomovitz BM, Drew Y, Arkenau HT, Machiels JP, Spicer JF, Jones R, Forster MD (2020). Tisotumab vedotin in previously treated recurrent or metastatic cervical cancer. Clin Can Res.

[152] Costantino V, Fattorusso E, Imperatore C, Mangoni A (2003). Ectyoceramide, the first natural hexofuranosylceramide from the marine sponge Ectyoplasia ferox. Eur J Org Chem.

[153] Costantino V, Fattorusso E, Imperatore C, Mangoni A (2008). Glycolipids from sponges. 20. J-coupling analysis for stereochemical assignments in furanosides: structure elucidation of vesparioside B, a glycosphingolipid from the marine sponge Spheciospongia vesparia. J Org Chem.

[154] Malve H (2016). Exploring the ocean for new drug developments: marine pharmacology. J Pharm Bioallied Sci.

[155] Jaspars M, De Pascale D, Andersen JH, Reyes F, Crawford AD, Ianora A (2016). The marine biodiscovery pipeline and ocean medicines of tomorrow. J Mar Biol Ass U K.

[156] Löwenberg B (2013). Sense and nonsense of high-dose cytarabine for acute myeloid leukemia. J Am Soc Hematol.

[157] Menis J, Twelves C (2011). Eribulin (Halaven): a new, effective treatment for women with heavily pretreated metastatic breast cancer. Breast Cancer.

[158] Klotz U (2006). Ziconotide-a novel neuron-specific calcium channel blocker for the intrathecal treatment of severe chronic pain-a short review. Int J Clin Pharm Ther.

[159] Sagar S, Kaur M, Minneman KP (2010). Antiviral lead compounds from marine sponges. Mar Drugs.

[160] Umeyama A, Matsuoka N, Mine R, Nakata A, Arimoto E, Matsui M, Shoji N, Arihara S, Takei M, Hashimoto T (2010). Polyacetylene diols with antiproliferative and driving Th1 polarization effects from the marine sponge Callyspongia sp. J Nat Med.

[161] Takei M, Umeyama A, Shoji N, Hashimoto T (2010). Polyacetylenediols regulate the function of human monocyte-derived dendritic cells. Int Immunopharmacol.

[162] Velmurugan BK, Lee CH, Chiang SL, Hua CH, Chen MC, Lin SH, Yeh KT, Ko YC (2018). PP2A deactivation is a common event in oral cancer and reactivation by FTY720 shows promising therapeutic potential. J Cell Phys.

[163] Lin Z, Antemano RR, Hughen RW, Tianero MDB, Peraud O, Haygood MG, Concepcion GP, Olivera BM, Light A, Schmidt EW (2010). Pulicatins A−E, neuroactive thiazoline metabolites from cone snail-associated bacteria. J Nat Prod.

[164] Asolkar RN, Freel KC, Jensen PR, Fenical W, Kondratyuk TP, Park EJ, Pezzuto JM (2009). Arenamides A− C, cytotoxic NFκB inhibitors from the marine actinomycete Salinispora arenicola. J Nat Prod.

[165] Ueoka R, Nakao Y, Kawatsu S, Yaegashi J, Matsumoto Y, Matsunaga S, Furihata K, Van Soest RW, Fusetani N (2009). Gracilioethers A−C, antimalarial metabolites from the marine sponge Agelas gracilis. J Org Chem.

[166] Martins A, Vieira H, Gaspar H, Santos S (2014). Marketed marine natural products in the pharmaceutical and cosmeceutical industries: tips for success. Mar Drugs.

[167] Molinski TF, Dalisay DS, Lievens SL, Saludes JP (2009). Drug development from marine natural products. Nat Rev Drug Dis.

[168] Montaser R, Luesch H (2011). Marine natural products: a new wave of drugs?. Future Med Chem.

